# Anisotropic Water Structure at Charged Interfaces Studied by Depth-Resolved Vibrational SFG/DFG Spectroscopy

**DOI:** 10.1021/jacs.5c19517

**Published:** 2026-04-16

**Authors:** Álvaro Díaz Duque, Vasileios Balos, Martin Wolf, Alexander P. Fellows, Martin Thämer

**Affiliations:** † 28259 Fritz-Haber-Institut der Max-Planck-Gesellschaft, Faradayweg 4-6, Berlin 14195, Germany; ‡ Instituto Madrileño de Estudios Avanzados En Nanociencia (IMDEA Nanociencia), Madrid 28049, Spain

## Abstract

The molecular water structure at charged aqueous interfaces is shaped by interfacial electric fields, which can induce significant anisotropy in the molecular orientations extending over nanometer-scale distances. Despite its great relevance, very little is known about the details of this depth-dependent anisotropic water structure, mainly due to the lack of appropriate experimental techniques. Here, we present a depth-resolved study of the water anisotropy at the interface with insoluble charged surfactants using a newly developed technique, which allows for directly correlating nonlinear vibrational spectra with depth information on the nanometer scale. We demonstrate that the obtained data allows for a reconstruction of the nonlinear vibrational responses as a function of depth. The results for the case of low-salinity solutions show the presence of two pronounced regions within the interfacial anisotropy with largely deviating degrees of preferential molecular orientations. A spectral analysis of the depth-dependent vibrational responses furthermore reveals that the natural local hydrogen-bond structure of bulk water remains largely unperturbed throughout the interfacial region, including water in direct proximity to the surface charges. These findings significantly refine our understanding of the anisotropic water structure at the interface with hydrophilic charged surfactants and showcase the large potential of our depth-resolved spectroscopic technique.

## Graphical abstract

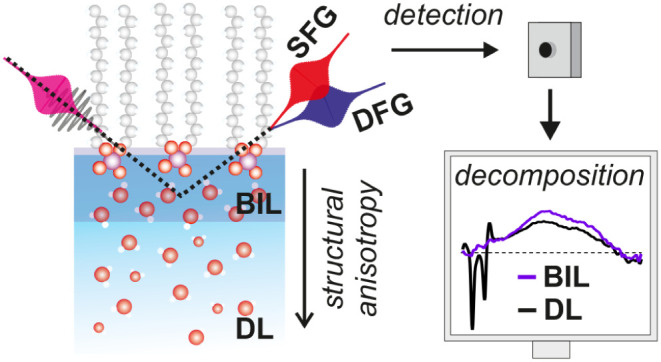

## Introduction

The elucidation of the depth-dependent molecular water structure at charged aqueous interfaces is a central goal in fundamental interface research because of its overwhelming relevance in a broad variety of fields, ranging from biophysics to environmental chemistry and electrochemistry.^1–6^ Particularly important for environmental chemistry are interfaces between water and charged surfactants as they represent the majority of the oceans surfaces and can also be found in aqueous aerosols.^7,8^ The presence of a net surface charge leads to reorientation of water molecules in the subsurface region and thus influences the interfacial molecular structure.^9–11^ This process is induced by the electrostatic field that penetrates the subsurface region and decays toward the bulk. The resulting preferential orientation makes the molecular structure anisotropic, which has important consequences for the thermodynamic properties of the interface as e.g., orientational ordering generally leads to a lowering of entropy.^12,13^ These ordering effects therefore have a crucial impact on many aspects of interfacial chemistry such as transport processes across the interface, macroscopic surface properties e.g., surface tension, as well as modulations of local electrochemical potentials.^14–19^

The thickness of the structurally anisotropic region is thereby highly variable and can reach up to hundreds of nanometers. The length-scale of the anisotropy decay depends, for example, on the salt concentration of the electrolyte that modulates the screening of the electrostatic field. At high ionic strengths, the anisotropy will decay faster as a function of depth than for low ionic strengths.^20^ Determining the exact evolution of the water structure with depth has, however, proven extremely challenging, both theoretically and experimentally.^21–26^ While the evolution of the electrostatic potential with depth is commonly described by diverse theoretical models such as Gouy–Chapman, Gouy–Chapman–Stern, and further refining modifications,^20,27^ these theories become increasingly inaccurate for higher potentials like those typically present very close to the surface charges. Furthermore, these theories do not provide direct information on the resulting anisotropic water structure as they treat water as a homogeneous medium. In consequence, our understanding of these structural details remains highly limited.

Over the last few decades, the water structure at charged interfaces has been intensively studied using computer simulations and experiments. However, the results do not provide a conclusive general picture of the molecular water structures in the subsurface region. Open questions include, for example, whether the water structure in direct contact with the surface charges is fundamentally different than the water structure in the layers below. Diverse studies present very contrasting views of the topmost water layers, ranging from nearly perfectly ordered molecular alignment^28^ to rather broad orientational distributions.^29^ Another recent study concluded that the net-orientation of water molecules in the first few layers can even be reversed in the layers below via overscreening.^30^ Interestingly, local capacitance measurements have shown a drastic drop in the dielectric constant (relative permittivity) of water normal to the interface in the vicinity of surface charges (from 80 down to values around 2).^31^ Since the dielectric constant in water is dominated by dipolar reorientation, this observation suggests a relatively rigid molecular structure in that region with few degrees of freedom for molecular rotations. Such a scenario makes it seem likely that the H-bond network also shows significant changes in strength, connectivity, and dynamics. A more detailed picture of the structural properties of water at charged interfaces is meanwhile still absent, which is largely due to the lack of a direct experimental probe of the depth-dependent water anisotropy.

An experimental technique that has been widely used to study water structures at charged aqueous interfaces is phase-sensitive vibrational sum frequency generation (SFG) spectroscopy.^22,26,32–38^ The benefits of this technique for such studies are 2-fold: the vibrational water spectra are highly sensitive to the hydrogen bond environment, with strong hydrogen bonds leading to red-shifted OH stretch resonances, and weak hydrogen bonds leading to blue-shifts.^39,40^ That way, vibrational water spectra allow for the characterization of the H-bond network. Second, due to the special selection rules governing the second-order light-matter interactions, the technique is sensitive to anisotropic molecular arrangements (within the electric dipole approximation).^41–44^ In SFG the sign of the response is directly related to the molecular orientation forming peaks or dips in the spectra.^45–47^ This property makes the signals from isotropic regions vanish (via cancellation), which allows for exclusively probing the regions possessing structural anisotropy, such as the interfacial water at charged interfaces. As the anisotropy in these aqueous systems is dominated by preferential molecular orientation, the second-order susceptibility, χ^(2)^, which is the quantity of interest in SFG spectroscopy, serves as a direct measure of its extent. A clear limitation of this technique in its traditional form is, however, that the measured response is the integration over the entire anisotropic region, thus the important evolution of the anisotropy with depth, χ^(2)^(*z*), is inaccessible.^48^ Acknowledging this deficiency, several advanced SFG approaches have been developed in recent years. Among these techniques are transmission/reflection SFG spectroscopy^23^ as well as angular dependent^22,49,50^ and momentum dependent^24^ SFG approaches. Other methods combine SFG spectroscopy with phase sensitive second harmonic generation spectroscopy (SHG) and measurements of the streaming potentials to retrieve the desired depth information.^26,51^ While these studies indeed provide further insight into the depth-dependent water structure they, in part, report contradictory results. This might be due to the intrinsically limited depth-sensitivity of the techniques^48^ and/or the requirements for extended theoretical models and the use of contentious assumptions, which make an unambiguous interpretation of the measurement results very challenging.

Recently, we have presented a new concept in second-order vibrational spectroscopy that inherently yields a combination of nonlinear vibrational spectra of molecular interfaces with depth information on the subnm scale.^48,52–54^ The technique is based on the simultaneous, phase-sensitive measurement of sum- and difference-frequency generation signals (SFG and DFG) and enables the decomposition of signal contributions from different depths within the interfacial region. The desired depth information is thereby encoded in the phase and amplitude differences between the resulting SFG and DFG spectra. Because of its capability to provide precise depth information, the use of this experimental approach to characterize the anisotropy at charged aqueous interfaces bears high potential. Nonetheless, such implementation is far from trivial. As the desired depth information must be extracted from small changes in relative phase and amplitude between SFG and DFG signals, extraordinary accuracy and precision of the measurement is required. Achieving this for measurements on aqueous interfaces that are constantly in motion and yield extremely small SFG signals is obviously highly challenging.

In this contribution, we present an SFG/DFG spectrometer that fulfills these requirements, and demonstrate its suitability for depth-resolved studies of charged aqueous interfaces by extracting and analyzing the vibrational responses of anisotropic water within the first nanometer of the interface and the region below. In the first part, we describe the theory underlying this technique, followed by a presentation of the experimental details of the approach. In the second part, we present depth-resolved measurements of aqueous electrolyte solutions with insoluble charged surfactants. From the data obtained in these measurements, we successfully reconstruct the depth-dependent second-order susceptibility quantitatively, which yields crucial insights into the depth-dependent molecular structure in these systems.

## Theory

The concept of depth-resolved nonlinear vibrational spectroscopy is based on the simultaneous measurement of phase-resolved SFG and DFG spectra from a sample of interest. While the basic principle of this concept is described elsewhere,^48,52,53,55^ here we present the full theoretical framework of this technique from an experimental perspective (main text and Supporting Information) which includes the mathematical and graphical description of the nonlinear light-matter interaction for ultrashort laser pulses.

In vibrational second-order spectroscopy, a nonlinear signal $\left({Ẽ}_{\left(\omega_{\rho }\right)}\right)$ is generated by the frequency-mixing of two input fields ${Ẽ}_{\left(\omega_{\mathrm{IR}}\right)}$ and ${Ẽ}_{\left(\omega_{\mathrm{vis}}\right)}$ . The response follows the general relation:^54,56^

1 $${Ẽ}_{\left(\omega_{\rho }\right)}\propto \int_{-\infty }^{\infty }{d\omega }_{\mathrm{IR}}\int_{-\infty }^{\infty }{d\omega }_{\mathrm{vis}}{Ẽ}_{\left(\omega_{\mathrm{IR}}\right)}{Ẽ}_{\left(\omega_{\mathrm{vis}}\right)}\chi_{\mathrm{eff},\left(\omega_{\rho }=\omega_{\mathrm{IR}}+\omega_{\mathrm{vis}}\right)}^{\left(2\right)}\cdot \delta \left(\omega_{\rho }-\omega_{\mathrm{IR}}-\omega_{\mathrm{vis}}\right)$$

where $\chi_{\mathrm{eff},\left(\omega_{\rho }=\omega_{\mathrm{IR}}+\omega_{\mathrm{vis}}\right)}^{\left(2\right)}$ is the effective second-order susceptibility. Because all fields are real in their time domain representation, their frequency axes span from negative to positive infinity. As $\chi_{\mathrm{eff},\left(\omega_{\rho }=\omega_{\mathrm{IR}}+\omega_{\mathrm{vis}}\right)}^{\left(2\right)}$ depends on two independent frequencies, it can be expressed in the form of a two-dimensional frequency plane. The overall integral in eq 1 can consequently be split into the following four contributions corresponding to the four quadrants in this 2D frequency plane (Figure 1a):

**Figure 1:**
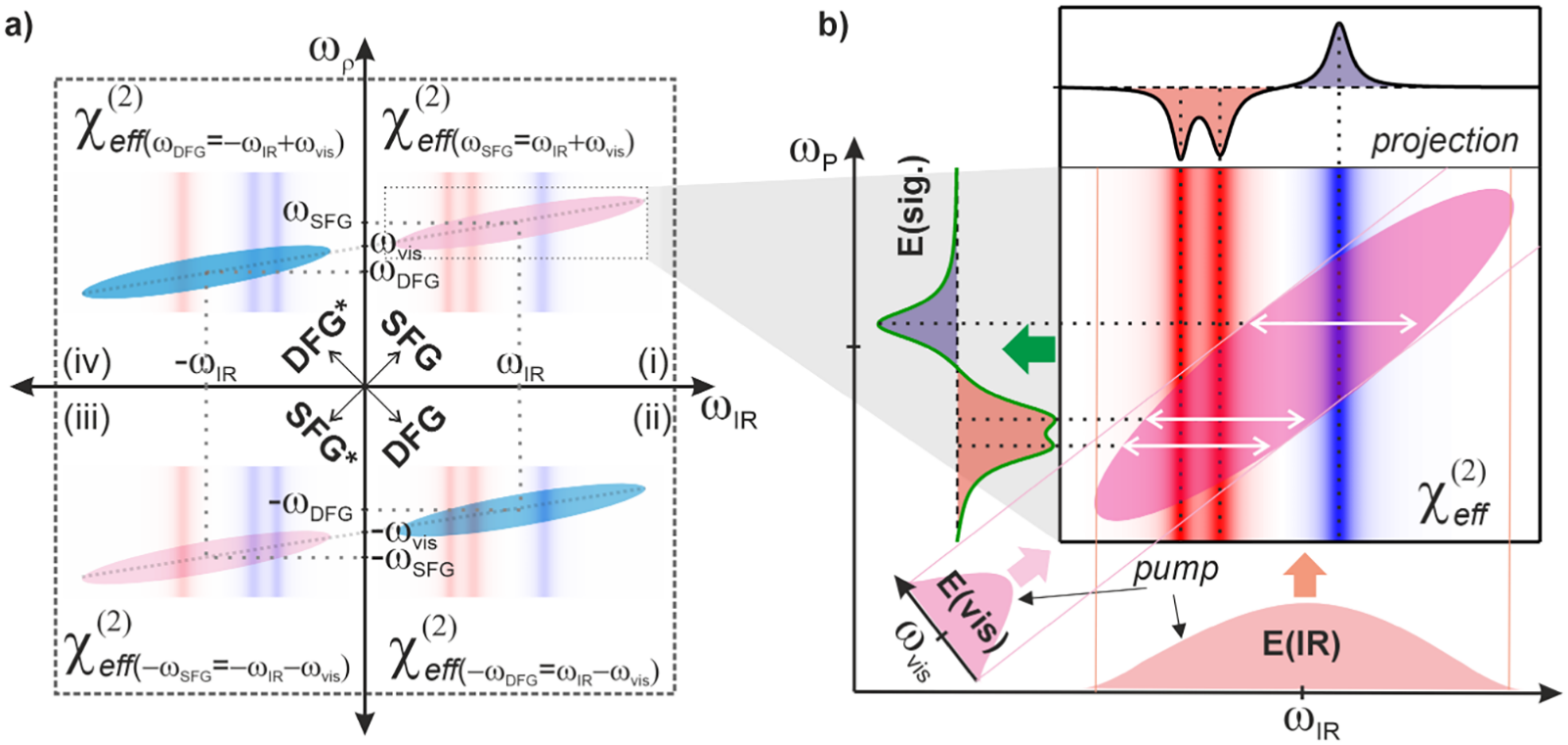
(a) Two-dimensional representation of the effective second-order susceptibility as a function of infrared and signal frequencies. SFG and DFG signals are represented by the pink and blue shaded areas, respectively. For better clarity only vibrational resonances are considered, being indicated by the vertical, colored lines. b) Graphical representation of second-order nonlinear interactions illustrated for the response in quadrant (i) in (a). The nonlinear signal *E*(sig.) is obtained by multiplication of the infrared pump field *E*(IR) and the visible field *E*(vis) with the nonlinear susceptibility. The spectrum on the top represents the projection of the susceptibility onto the vibrational axis. The spectrum of *E*(sig.) shows the same vibrational resonance features as the projection but with broadened line shape which originates from the bandwidth of the visible upconversion pulse used in this example (see Supporting Information for further details). For simplicity, only the imaginary parts of the susceptibility and the electromagnetic fields are shown in the figure.

Quadrant (i) in Figure 1a

2 $${Ẽ}_{\left(\omega_{\mathrm{SFG}}\right)}\propto \int_{0}^{\infty }{d\omega }_{\mathrm{IR}}\int_{0}^{\infty }{d\omega }_{\mathrm{vis}}{Ẽ}_{\left(\omega_{\mathrm{IR}}\right)}{Ẽ}_{\left(\omega_{\mathrm{vis}}\right)}\chi_{\mathrm{eff},\left(\omega_{\rho }=\omega_{\mathrm{IR}}+\omega_{\mathrm{vis}}\right)}^{\left(2\right)}\cdot \delta \left(\omega_{\rho }-\omega_{\mathrm{IR}}-\omega_{\mathrm{vis}}\right)$$

Quadrant (iii) in Figure 1a

3 $${Ẽ}_{\left(-\omega_{\mathrm{SFG}}\right)}\propto \int_{-\infty }^{0}{d\omega }_{\mathrm{IR}}\int_{-\infty }^{0}{d\omega }_{\mathrm{vis}}{Ẽ}_{\left(\omega_{\mathrm{IR}}\right)}{Ẽ}_{\left(\omega_{\mathrm{vis}}\right)}\chi_{\mathrm{eff},\left(\omega_{\rho }=\omega_{\mathrm{IR}}+\omega_{\mathrm{vis/mi>}}\right)}^{\left(2\right)}\cdot \delta \left(\omega_{\rho }-\omega_{\mathrm{IR}}-\omega_{\mathrm{vis}}\right)$$

Quadrant (ii) in Figure 1a

4 $${Ẽ}_{\left(-\omega_{\mathrm{DFG}}\right)}\propto \int_{0}^{\infty }{d\omega }_{\mathrm{IR}}\int_{-\infty }^{0}{d\omega }_{\mathrm{vis}}{Ẽ}_{\left(\omega_{\mathrm{IR}}\right)}{Ẽ}_{\left(\omega_{\mathrm{vis}}\right)}\chi_{\mathrm{eff},\left(\omega_{\rho }=\omega_{\mathrm{IR}}+\omega_{\mathrm{vis}}\right)}^{\left(2\right)}\cdot \delta \left(\omega_{\rho }-\omega_{\mathrm{IR}}-\omega_{\mathrm{vis}}\right)$$

Quadrant (iv) in Figure 1a

5 $${Ẽ}_{\left(\omega_{\mathrm{DFG}}\right)}\propto \int_{-\infty }^{0}{d\omega }_{\mathrm{IR}}\int_{0}^{\infty }{d\omega }_{\mathrm{vis}}{Ẽ}_{\left(\omega_{\mathrm{IR}}\right)}{Ẽ}_{\left(\omega_{\mathrm{vis}}\right)}\chi_{\mathrm{eff},\left(\omega_{\rho }=\omega_{\mathrm{IR}}+\omega_{\mathrm{vis}}\right)}^{\left(2\right)}\cdot \delta \left(\omega_{\rho }-\omega_{\mathrm{IR}}-\omega_{\mathrm{vis}}\right)$$

where the first two correspond to SFG responses, and the other two to the DFG responses (as indicated in Figure 1a), representing complex conjugate pairs. Importantly, the measured SFG and DFG responses are not generally equal, with their relation depending on the resonance conditions and spatial origin of the nonlinear signals, as discussed later. Following the two-dimensional representation of the second-order interaction, the mathematical description of the nonlinear response can be visualized graphically. The nonlinear interaction of the material with the IR beam (included in the Figure 1b) corresponds to a multiplication of each row of the susceptibility matrix with the complex spectrum of the IR pulses (vertical interaction). Because of the relation ω_ρ_ = ω_IR_ + ω_vis_, the nonlinear interaction with the visible beam appears along the diagonal (linearly shifted by the visible center frequency). The multiplication of the matrix with the complex spectrum of the visible beam along this diagonal now generates a tilted region in the matrix (indicated by the pink, oval-shaped region in Figure 1b) that contributes to the nonlinear response. The latter is obtained by simple projection of this region onto the vertical (ω_ρ_) axis. This graphical representation is general and fully describes the operations in eq 1. It can even include cases with electronic resonances and vibronic coupling, although this is not shown in Figure 1b for clarity. The clear benefit of this graphical representation is that it yields a straightforward way of visualizing the nonlinear interactions which lead to the generation of the second-order signals e.g., for comparisons of the different experimental approaches to obtain the desired nonlinear vibrational information as shown in the Supporting Information.

The goal of typical SFG experiments is to extract the vibrational resonance information, which can be done using several spectroscopic approaches as discussed in the Supporting Information. The obtained vibrational spectra then allow for identification of molecular species and the elucidation of molecular structures. Besides the resonant information, the two-dimensional effective susceptibility represented in Figure 1a also contains the desired depth information. The origin of this lies in the connection between the measured effective susceptibility and the desired depth-dependent local susceptibility χ^(2)^(*z*), a 3-dimensional quantity) which is given by the following equation

6 $$\chi_{\mathrm{eff},\left(\omega_{\rho }=\omega_{\mathrm{IR}}+\omega_{\mathrm{vis}}\right)}^{\left(2\right)}=\int_{0}^{\infty }{d\mathrm{zL}}_{\left(\omega_{p}\right)}L_{\left(\omega_{\mathrm{vis}}\right)}L_{\left(\omega_{\mathrm{IR}}\right)}\chi^{\left(2\right)}\left(z\right)e^{i\Delta k_{z}z}$$

where $L_{\left(\omega_{i}\right)}$ are the nonlinear Fresnel factors and Δ*k*_*z*_ is the *z*‑component of the wavevector mismatch $(\Delta k_{z}^{\mathrm{SFG}}=\left|k_{z}\left(\omega_{\mathrm{IR}}\right)\right|+\left|k_{z}\left(\omega_{\mathrm{vis}}\right)\right|+\left|k_{z}\left(\omega_{\rho }\right)\right|$ and $\Delta k_{z}^{\mathrm{DFG}}=\left|k_{z}\left(\omega_{\mathrm{IR}}\right)\right|-\left|k_{z}\left(\omega_{\mathrm{vis}}\right)\right|-\left|k_{z}\left(\omega_{\rho }\right)\right|$ for positive ω_IR_ in the commonly applied reflection geometry, i.e., quadrants (i) and (ii) in Figure 1a, respectively). In typical applications of SFG or DFG, the frequencies are chosen such that the IR beam is resonant with some specific vibration of the sample while the visible frequencies are fully nonresonant.^43,57^ In such a case, the intrinsic second-order susceptibility (χ^(2)^(*z*)) becomes largely independent of the visible and output frequencies^54^ and can be, in good approximation, replaced by $\chi_{\left(\omega_{\mathrm{IR}}\right)}^{\left(2\right)}\left(z\right)$ that only depends on IR frequencies. In fact, it is important to note that the quantity of interest in a vibrational SFG experiment (the “SFG spectrum”) is the spectrum along this vibrational axis of the second-order susceptibility, and not the spectrum of the generated SFG light. Applying the nonresonant condition at visible frequencies to χ^(2)^(*z*), we obtain two-dimensional frequency planes with the IR resonances along the horizontal dimension while all the values remain constant along the vertical axis (as indicated in Figure 1a and b). As a result, the SFG and DFG responses in each $\chi_{\left(\omega_{\mathrm{IR}}\right)}^{\left(2\right)}\left(z\right)$ plane that correspond to the same side of the vibrational axis become equal.^54^ However, such equality is still not generally given for the measured responses (effective susceptibility, $\chi_{\mathrm{eff},\left(\omega_{\rho }=\omega_{\mathrm{IR}}+\omega_{\mathrm{vis}}\right)}^{\left(2\right)}$ ). As shown in eq 6, the χ^(2)^(*z*) is modulated by the phase factor $e^{i\Delta k_{z}z}$ , which introduces depth-dependent phase shifts to the generated signals. These phase shifts are different for SFG and DFG in magnitude and direction. The discrepancy originates from the different values of Δ*k*_*z*_ (different sign and magnitude) for SFG and DFG signals as individual *k* values invert their sign for regions with negative frequency in the two-dimensional representation of $\chi_{\mathrm{eff},\left(\omega_{\rho }=\omega_{\mathrm{IR}}+\omega_{\mathrm{vis}}\right)}^{\left(2\right)}$ . This makes the effective SFG and DFG responses sensitive to the spatial origin (depth) of the signal, with significant deviations between SFG and DFG spectra for signals from larger depths and overlapping spectra for pure surface signals. Particularly sensitive to depth is thereby the phase difference between the SFG and DFG spectra with ca. 2 deg. per nm.^52^ This property can be exploited to extract the desired depth information, as shown in the result section.

## Experimental Section

For a successful depth-resolved study, the phase-resolved SFG and DFG spectra need to be obtained at very high accuracy and precision, which places significant requirements for the experimental implementation of such a spectroscopy. The two signals (SFG and DFG) are generated from the same nonlinear process, meaning that every SFG response is always accompanied by a corresponding DFG signal.^42,48,56,58^ In consequence, each SFG experiment can in principle be upgraded to a SFG/DFG spectrometer by simply detecting the simultaneously generated DFG signal. Phase-resolution for each of the two responses can then be achieved interferometrically by superimposing the nonlinear signals with corresponding reference beams, so-called local oscillators (LO).^59,60^ In consequence, any of the common experimental SFG methods such as narrow- and broadband frequency-domain approaches^61,62^ as well as the recently introduced fully broadband time-domain method^59,60^ can in principle be used. However, these techniques are not equally suitable for high precision phase-resolved SFG/DFG experiments on aqueous interfaces. As detailed in the Supporting Information, only the broadband time-domain method presented here allows for combining the highly beneficial collinear beam geometry with an efficient suppression of parasitic signal contributions (which is a fundamental requirement for such measurements). The concept of this time-domain approach for phase-resolved SFG spectroscopy was published earlier,^52^ here we describe the experimental details of the extension of this concept to an SFG/DFG spectrometer.

Midinfrared and visible (690 nm) pulses are generated from a commercial 1 kHz Ti:Sa laser system (800 nm; <35 fs pulses) including two independent optical parametric amplifiers (see Supporting Information). Inside the spectrometer (see Figure 2), a small portion of the IR pulse is split out from the main beam path using a KBr beamsplitter. This weak portion is used for the generation of the local oscillators and passes through a pair of free-standing wire grid polarizers (GP-1 and -2) for power control. Subsequently, this beam portion is collinearly overlapped with the visible on a customized incoupling optic (ICO-1). These two pulses are then focused on a thin (20 μm) z-cut quartz crystal where the SFG and DFG LOs are generated (for details see Supporting Information), which then travel collinearly with the visible pulse. After passing a delay stage, these pulses are overlapped collinearly (ICO-2) with the main portion of the IR and leave the interferometer.

**Figure 2:**
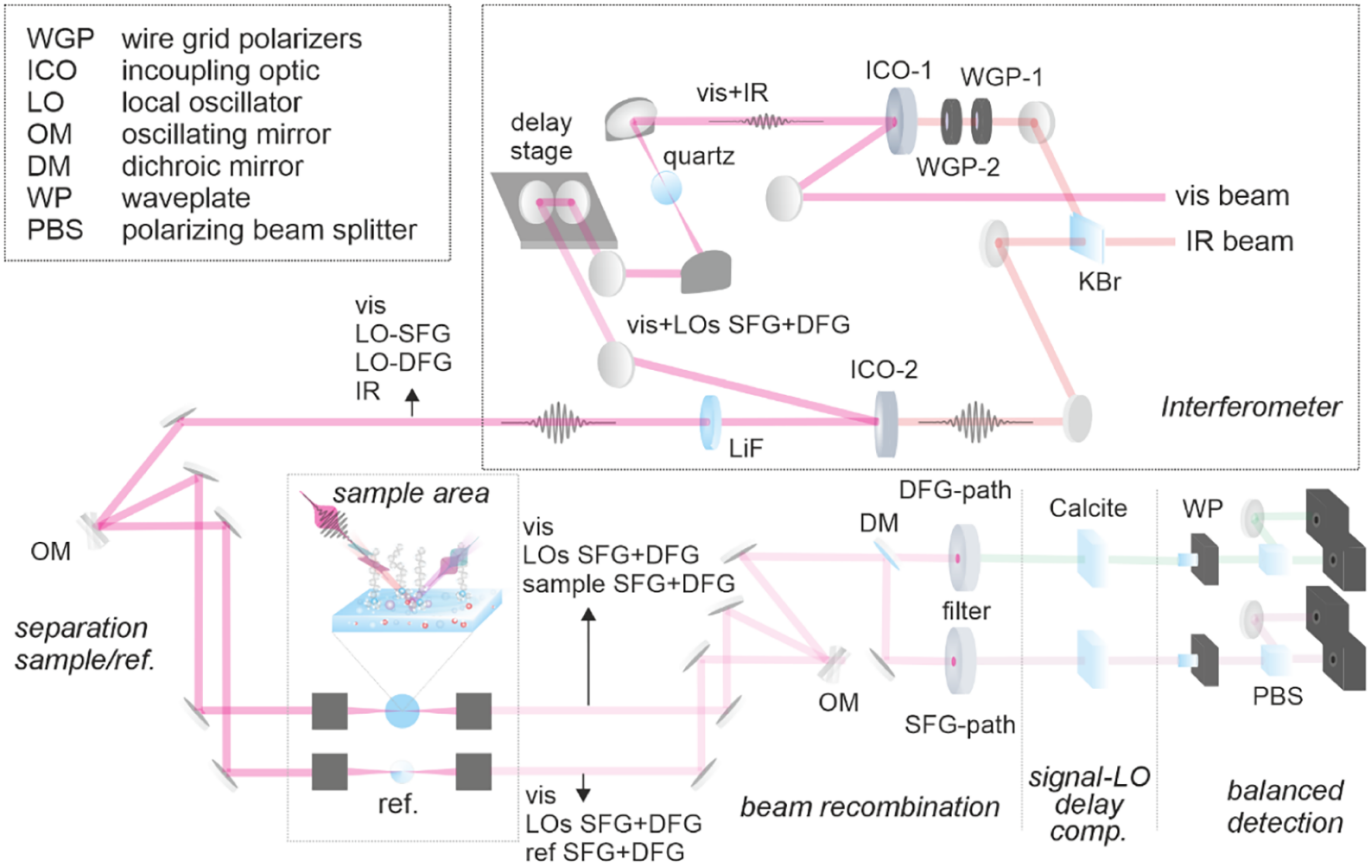
Schematic of the fully collinear phase-resolved SFG and DFG spectrometer.

The single beam output of the interferometer contains four pulses, namely the IR and visible pulses, as well as SFG and DFG LOs, and is guided to the sample area. Before reaching the sample, the beam is split into two portions using a 500 Hz oscillating galvo mirror (OM). The two resulting beams are independently focused on the sample and a reference with off-axis parabolic mirrors (OPMs). Using this approach, every other laser pulse probes sample and reference, which allows for efficient suppression of any phase drifts over the course of the experiment.^52^ Just before reaching the sample and reference interfaces, the beams pass through LiF windows for the removal of parasitic signal contributions (see Supporting Information). The generated SFG/DFG signals, as well as the respective LOs, which are linearly reflected at the sample surface, are collimated by a second set of OPMs and the two beam-paths are recombined on the same oscillating galvo mirror and sent to detection. A dichroic beam splitter separates SFG and DFG frequencies and sets of edge-pass filters isolate the two heterodyned nonlinear signals. At this point, LOs and nonlinear signals are orthogonally polarized.^60^ A pair of calcite crystals is used for tuning the temporal overlap of the nonlinear signals with their respective LOs (details are given in the Supporting Information). After a combination of waveplates (WPs) and polarizing beam splitters (PBSs), the interference cross-terms between LOs and nonlinear signals are recorded in a balanced detection scheme,^60^ for both the SFG and DFG responses.

During a measurement, the delay between the IR and the combined visible and LOs pulses is scanned by moving the delay stage at a constant speed, while recording the resulting intensity on the detectors for each laser pulse. Scanning the delay stage modulates phase and amplitude of SFG and DFG signals with respect to their LOs, yielding separate interferograms. Due to the alternating probing of reference and sample, we obtain four interferograms for each scan, namely SFG and DFG from both the sample and reference. The final phase-resolved spectra are obtained by Fourier transformation of the sample responses and normalization to the reference responses. Using this time-domain interferometric approach we obtain nonlinear vibrational spectra that correspond to the projection of the two-dimensional susceptibility onto the vibrational frequency axis shown in Figure 1b (for more details see Supporting Information).

## Results

With this spectroscopic setup in hand, we turn to depth-resolved measurements of interfacial water at the boundary with insoluble charged surfactants. The surfactants employed are dihexadecyldimethylammonium bromide (DHAB) and dihexadecyl phosphate (DHP), which present positively and negatively charged headgroups, respectively, and form densely packed monolayers at the aqueous interface.^63,64^ The polarity of the surface charges dictates the water orientation with the molecular dipoles preferentially pointing up for negative and pointing down for positive charges. The resulting anisotropic orientational distribution of water molecules should therefore give rise to SFG/DFG responses with positive (negative) imaginary parts for a net dipole orientation pointing up (down).

Figure 3 shows the imaginary parts of the obtained SFG (red traces) and DFG (blue traces) spectra for aqueous interfaces with DHP (negatively charged, left-side) and DHAB (positively charged, right side), measured in SSP polarization. All the spectra exhibit, at their lower frequencies (below 3000 cm^–1^), pronounced resonant features that correspond to the CH stretching modes from the terminal methyl groups of the surfactant tails. At higher frequencies, there is a broad absorption band between 3000 and 3600 cm^–1^, which corresponds to the OH stretch vibration of the interfacial water molecules with an anisotropic orientational distribution.^65^ As expected, a clear sign flip of the OH stretching response can be observed in the spectra when replacing the negative charges with positive charges, confirming the orientational flip of the water molecules for the two different charge polarities.^66^ In contrast, for both negatively and positively charged surfactants, the sign of the CH stretching bands remains unchanged. This behavior is expected, since the terminal methyl groups point in the same direction in both systems.

**Figure 3:**
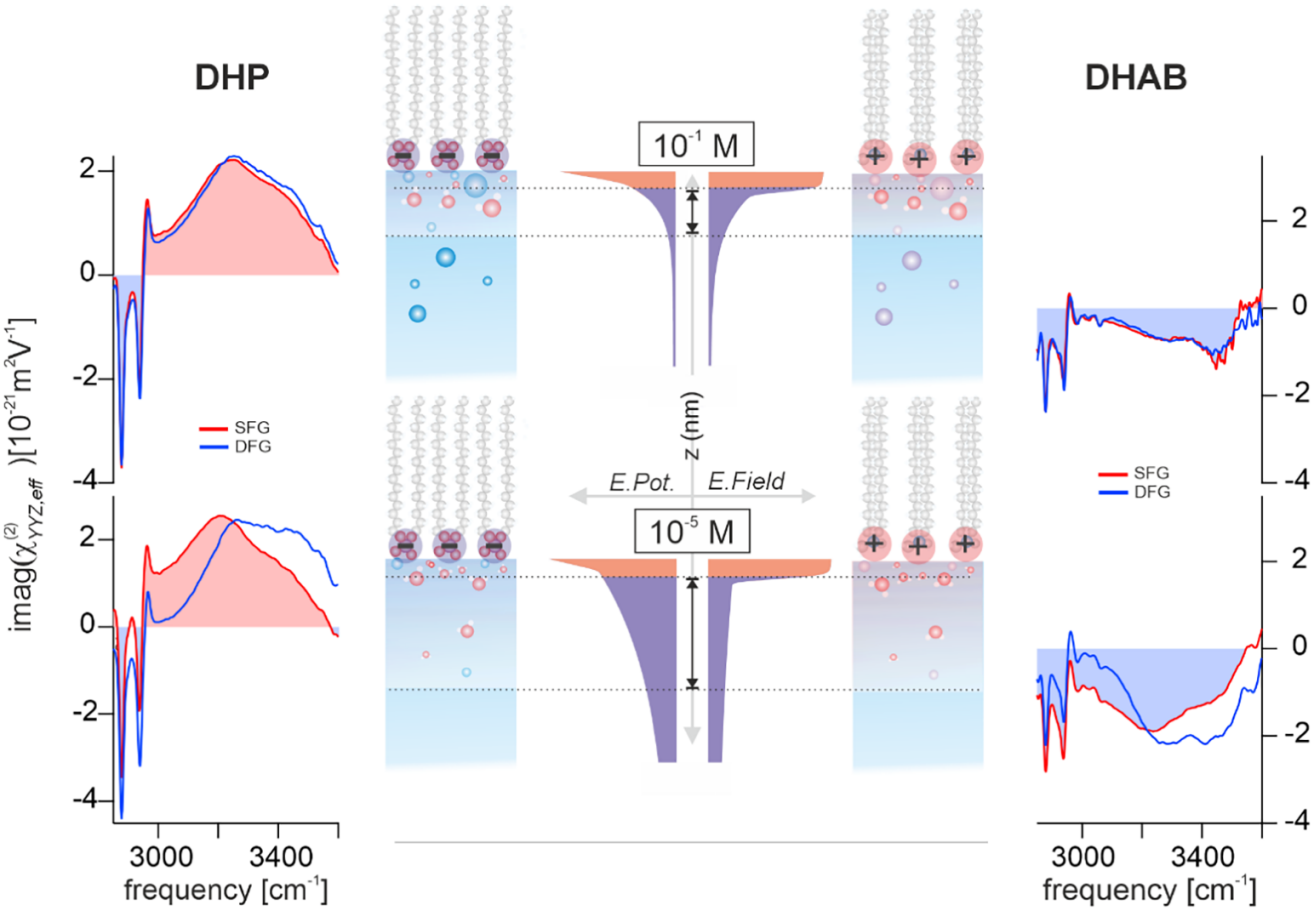
Imaginary SFG (red) and DFG (blue) spectra of charged aqueous interfaces with DHP (dihexadecyl phosphate, negatively charged, left) and DHAB (dihexadecyldimethylammonium bromide, positively charged, right) at 10^–1^ M (upper) and 10^–5^ M (lower) NaCl concentrations. Middle panel: schematics of the evolution of the electric potential and field as predicted by the GCS model shown for both salt concentrations. Lower amplitudes in the spectra of the DHAB samples (CH and OH region) indicate a lower surface coverage of DHAB as compared to DHP. Small deviations between SFG and DFG spectra of the 10^–1^ M solutions originate from magnetic dipole contributions as detailed in the Supporting Information.

The SFG/DFG measurements of these charged aqueous interfaces are performed at two different salt concentrations in the aqueous solution, with the two samples in the upper panel at 10^–1^ M (NaCl), and the solutions in the lower panel at 10^–5^ M (NaCl). In the case of high concentration, the electric field from the charges is largely screened and decays on a length scale of approximately 1 nm (Debye length). In consequence, the spatial extent of the water anisotropy should be confined within this thin region. The expected small thickness of the anisotropic layer is clearly confirmed by the significant spectral overlap of SFG and DFG spectra (no considerable phase or amplitude difference between the spectra). In contrast, the field screening in the low concentration case is much weaker with the result that the field reaches much further into the bulk and decays on a length scale of ca. 96 nm.^27^ The largely increased thickness of the water layer with preferential molecular orientation in these samples now gives rise to the strong deviations between the SFG and DFG line shapes as shown in the lower panels of Figure 3.

The presented measurements clearly confirm the expected behavior of the depth-dependent structural anisotropy under different salinities and demonstrate the sensitivity of the SFG/DFG spectroscopy to these changes in the spatial extent of the anisotropic region. However, for a detailed analysis of the evolution of the structural anisotropy of interfacial water with depth, the depth-dependent susceptibility $\chi_{\left(\omega_{\mathrm{IR}}\right)}^{\left(2\right)}\left(z\right)$ needs to be determined from these measurements. In order to do so, we turn to the experiment with negatively charged (phosphate) surfactants at low salt concentrations (bottom left in Figure 3) and use a description of the depth-dependent electric potential adopted from the Gouy–Chapman–Stern theory (GCS).^27^ The GCS model predicts two electrostatic regimes: a very thin layer directly at the phase boundary, where the electric potential largely drops over the first few water layers (bonded interfacial layer, BIL), followed by an extended region where the potential decays exponentially (diffuse layer, DL). The decay length of the potential in the diffuse layer is thereby given by the Debye length *z*_DL_. The generated electrostatic field is then given by the negative derivative of the potential and will be relatively large within the BIL followed by a much smaller and exponentially decaying field in the DL (with the same decay constant as for the potential). The amount of preferential molecular orientation of water will somehow follow the evolution of the field and we can consequently also expect $\chi_{\left(\omega_{\mathrm{IR}}\right)}^{\left(2\right)}\left(z\right)$ to consist of two distinct regimes, $\chi_{\mathrm{BIL}}^{\left(2\right)}\left(z\right)$ and $\chi_{\mathrm{DL}}^{\left(2\right)}\left(z\right)$ .^67,68^ Within the DL, water is exposed to a relatively weak electrostatic field, and we can therefore safely assume that the local hydrogen bond structure of water in this region is not altered. In this weak-field region, the $\chi_{\mathrm{DL}}^{\left(2\right)}\left(z\right)$ should be proportional to the electrostatic field and decay exponentially with the Debye length *z*_DL_.^21,32,69^

The exact relation between the electrostatic field and $\chi_{\left(\omega_{\mathrm{IR}}\right)}^{\left(2\right)}\left(z\right)$ within the BIL is much less clear due to the much higher field strength and potential modifications of the hydrogen bond structure by the solvation of the surfactants headgroups. The observed drastic change in dielectric constant in the topmost water layers clearly suggests a highly nonlinear relation within the BIL. However, because of the small thickness of the BIL (we assume this layer to be approximately 1 nm thick), we can describe the evolution of the susceptibility with depth in the BIL by an effective response represented by $\chi_{\mathrm{BIL}}^{\left(2\right)}$ . The overall depth-dependent susceptibility can therefore be described by the following equation:

7 $$\chi_{\left(\omega_{\mathrm{IR}}\right)}^{\left(2\right)}\left(z\right)=\{\begin{array}{ll} \chi_{\mathrm{BIL}}^{\left(2\right)} & 0<z<z_{\mathrm{BIL}}\\ \chi_{\mathrm{DL}}^{\left(2\right)}\cdot e^{-\left(z-z_{\mathrm{BIL}}\right)/z_{\mathrm{DL}}} & z\geq z_{\mathrm{BIL}} \end{array}$$

At this point it is important to note that this notation does not include any assumptions on the depth-dependent evolution of the susceptibility within the BIL. The quantity $\chi_{\mathrm{BIL}}^{\left(2\right)}$ in the equation above represents the average response of this ca. 1 nm thick region. Furthermore, the DL contribution in eq 7 is often described in the literature by a DC field-induced $\chi^{\left(3\right)}$ response, i.e., $\chi_{\mathrm{DL}}^{\left(3\right)}E_{\mathrm{DC}}$ .^69–71^ The description used here is in principle equivalent with this more common notation but expresses the response in terms of the anisotropic structure resulting from the field-induced reorientation, i.e., the second-order response $\chi_{\mathrm{DL}}^{\left(2\right)}$ . We believe that this choice better highlights the fact that SFG directly probes the structural anisotropy of water rather than the DC field.

Inserting eq 7 into eq 6 yields following results for the predicted SFG and DFG spectra (exploiting the relation *z*_BIL_ ≪ Δ*k*_*z*_):

8 $$\chi_{\mathrm{SFG},\mathrm{eff}}^{\left(2\right)}=\chi_{\mathrm{BIL}}^{\left(2\right)}z_{\mathrm{BIL}}+\chi_{\mathrm{DL}}^{\left(2\right)}z_{\mathrm{DL}}\cdot C_{\mathrm{SFG}}\left(z_{\mathrm{DL}},{\Delta k}_{z}^{\mathrm{SFG}}\right)\cdot e^{i\cdot \mathrm{atan}\left({\Delta k}_{z}^{\mathrm{SFG}}\cdot z_{\mathrm{DL}}\right)}$$

9 $$\chi_{\mathrm{DFG},\mathrm{eff}}^{\left(2\right)}=\chi_{\mathrm{BIL}}^{\left(2\right)}z_{\mathrm{BIL}}+\chi_{\mathrm{DL}}^{\left(2\right)}z_{\mathrm{DL}}\cdot C_{\mathrm{DFG}}\left(z_{\mathrm{DL}},{\Delta k}_{z}^{\mathrm{DFG}}\right)\cdot e^{i\cdot \mathrm{atan}\left({\Delta k}_{z}^{\mathrm{DFG}}\cdot z_{\mathrm{DL}}\right)}$$

with $C\left(z_{\mathrm{DL}},\Delta k_{z}\right)=1/\sqrt{1+{z_{\mathrm{DL}}}^{2}\Delta {k_{z}}^{2}}$ . Note, the nonlinear Fresnel factors from eq 6 are here absorbed into the effective susceptibilities (see Supporting Information for details). From the equations it becomes clear that the BIL contributes equally to SFG and DFG responses, while the DL contribution deviates in phase and amplitude due to the opposite sign and different magnitude of Δ*k*_*z*_.

In consequence, the BIL contribution cancels in the difference between the measured SFG and DFG spectra, which allows for the isolation of the DL contribution. The resulting residual complex difference spectrum is given by

10 $$\Delta \chi_{\mathrm{eff}}^{\left(2\right)}=\chi_{\mathrm{DL}}^{\left(2\right)}z_{\mathrm{DL}}\left(C_{\mathrm{SFG}}\left(z_{\mathrm{DL}},{\Delta k}_{z}^{\mathrm{SFG}}\right)\cdot e^{i\cdot \mathrm{atan}\left({\Delta k}_{z}^{\mathrm{SFG}}\cdot z_{\mathrm{DL}}\right)}-C_{\mathrm{DFG}}\left(z_{\mathrm{DL}},{\Delta k}_{z}^{\mathrm{DFG}}\right)\cdot e^{i\cdot \mathrm{atan}\left({\Delta k}_{z}^{\mathrm{DFG}}\cdot z_{\mathrm{DL}}\right)}\right)$$

The term in brackets is unitless and depends on two key parameters: the wavevector mismatch Δ*k*_*z*_ and the Debye length *z*_DL_, both of which can be accurately calculated. For the given experimental geometry we obtain for ${\Delta k}_{z}^{\mathrm{SFG}}=0.021\;{\mathrm{nm}}^{-1}$ and for ${\Delta k}_{z}^{\mathrm{DFG}}=-0.013\;{\mathrm{nm}}^{-1}$ and for a NaCl concentration of 10^–5^ M the Debye length is *z*_DL_ = 96 nm. A simple division of the difference spectrum (difference between the SFG and DFG raw spectra) by the obtained complex value for this term yields the integrated DL spectrum. From this result, the effective DL contribution to the SFG (DFG) spectrum can be calculated using eq 8 ([Disp-formula eq9]), which can then be subtracted from the measured SFG (DFG) spectrum to yield the BIL contribution. With this simple procedure, both the DL and the BIL spectra can be isolated and, using eq 7, the entire evolution of the second-order susceptibility with depth can be reconstructed (a detailed description of this data analysis including a discussion on the accuracy and the limits of this approach are shown in the Supporting Information).

Figure 4 shows the decomposition of the measured spectra into the different contributions, namely BIL, DL, and the small residual component that originates from magnetic dipole transitions, MD.^72^ For the purpose of the presented analysis the MD signals represent a parasitic contribution which is consequently removed from the BIL and DL spectra (a more detailed description of the MD contributions and their experimental isolation is given in the Supporting Information). The decomposition shows that the measured spectra consist of a common contribution from the BIL (black trace lower panel), while the contributions from the DL are clearly phase-shifted and differ for SFG and DFG due to the opposite direction of the phase rotation. The contributions from the MD are meanwhile small and mainly contribute to the CH resonances.

**Figure 4:**
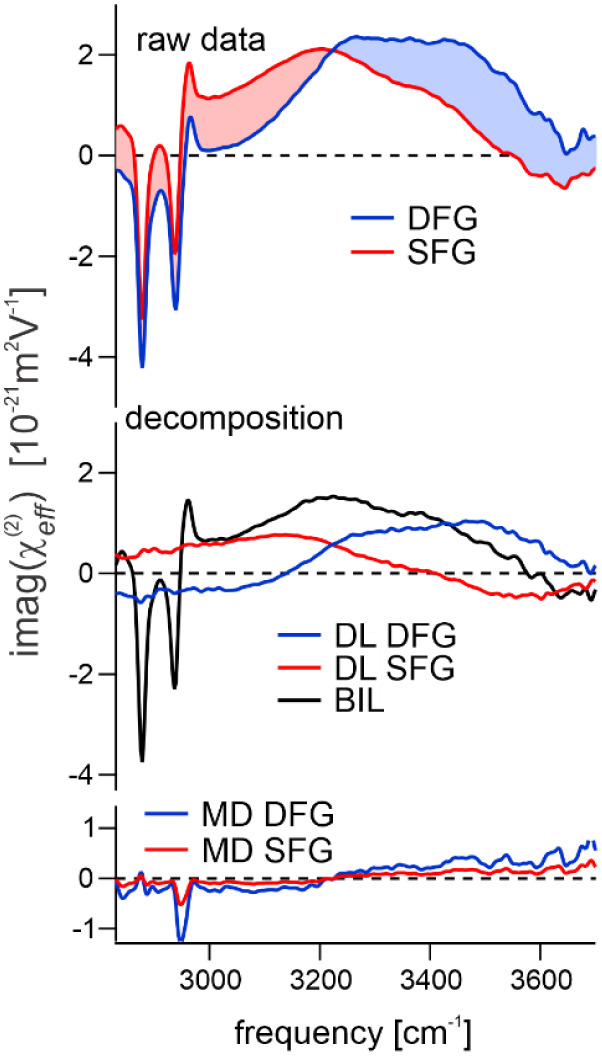
Decomposition of the measured SFG (red) and DFG (blue) spectra (DHP, *c*(NaCl) = 10^–5^ M) into the different effective contributions using the described method. Upper panel shows raw data, middle panel shows the contribution from the BIL (black, equal for SFG and DFG), and the corresponding DL contributions. The lower panel shows residual SFG and DFG contributions that originate from magnetic dipole transitions (see Supporting Information).

In order to compare DL and BIL spectra, the effect of the depth-related phase-shifts in the DL spectra must be removed, which can be done using eqs 8 and 9. The resulting spectra are shown in Figure 5a. The BIL spectrum (purple) represents the nonlinear vibrational response from the first approximately 1 nm of the sample $\left(\int_{o}^{z_{\mathrm{BIL}}}\chi_{\mathrm{BIL}}^{\left(2\right)}\left(z\right)\right)$ , while the DL spectrum (black) shows the nonlinear vibrational response beyond this near-surface region $\left(\int_{z_{\mathrm{BIL}}}^{\infty }\chi_{\mathrm{DL}}^{\left(2\right)}\left(z\right)\right)$ . A clear discrepancy between the two spectra is the fact that the resonant features from the surfactants (CH_3_ resonances) only appear in the BIL spectra. This intuitively follows expectations since the surfactant is highly insoluble in water and therefore only decorates the water surface. Beyond this, both spectra show a broad resonance peak centered at around 3200 cm^–1^ which corresponds to the O–H stretch vibration of anisotropic water. Importantly, these water peaks have a positive sign in both, BIL and DL, which shows that also the preferential molecular orientation in both regions points in the same direction. In consequence, there are no indications for an anomalous electrostatic effect such as overscreening at these interfaces. A more detailed comparison of these two water spectra shows surprising similarities in line-shape. Because of the strong dependency of the O–H stretching frequency on number and strength of H-bonds, this spectral similarity is clear evidence that, despite the proximity of these water molecules to the charged head groups, the H-bond environment is not significantly altered. On the contrary, it appears to be comparable to the local H-bond structure in the DL and thus similar to bulk water. In other words, there is no clear indication of any effect of the surface charges on the local H-bond structure (H-bond strength and connectivity) of the water layers just below the surface. The same seems to be true for the dynamics of the H-bond network which is well-known to highly influence the vibrational line shape of the O–H stretch vibration as well.^73–75^ The apparent similarity of the spectral line shapes suggests that the dynamics of the H-bond network in the BIL is at least not fundamentally different than in the DL.

**Figure 5:**
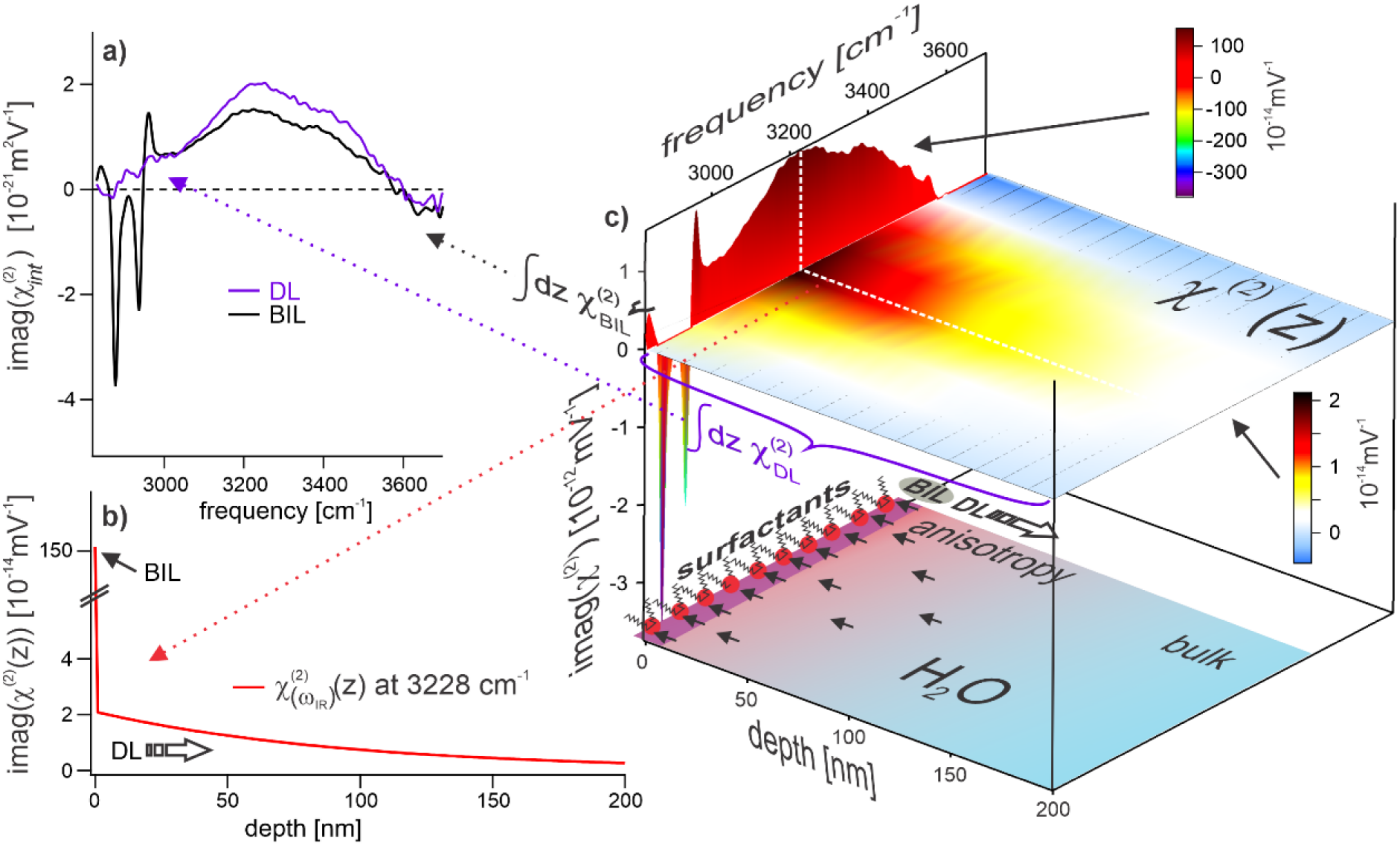
Depth-resolved second-order susceptibility at the charged water interface (DHP, *c*(NaCl) = 10^–5^ M). (a) Imaginary parts of the depth-integrated susceptibilities for BIL (black) and DL (purple), respectively. (b) Evolution of the susceptibility with *z* evaluated at the peak maximum of the water band (3228 cm^–1^). (c) Reconstructed 3D representation of the depth-dependent susceptibility (imaginary part). Note, the spectral contribution inside the thin BIL represents the average spectrum within this layer and consequently does not show any *z*-dependence.

Another interesting aspect of the obtained spectra is their evolution at high vibrational frequencies (>3600 cm^–1^), where the spectra turn to negative values. Such a negative spectral contribution at high frequencies had already been observed in the BIL spectra in a recent publication^65^ and was assigned to the response from isolated water molecules embedded in the hydrophobic tails of the surfactants. As those water molecules, if present, would not be H-bonded their vibrational response would indeed be shifted to higher frequency. Equally, due to their location above the headgroup charges, their orientation would also likely be opposite to the subsurface water, yielding the opposite sign SFG response. These expectations are hence in good agreement with the observed feature. However, our spectra clearly show that this negative spectral feature is equally present in the DL spectrum. In fact, both spectra highly overlap in that region. Since there is obviously no such hydrophobic region in the DL, the assignment of the spectral feature to such isolated water molecules no longer holds. It rather seems that the negative high frequency tail is part of the intrinsic SFG line shape of water in the presence of an electrostatic field, possibly resulting from a phase-shifted nonresonant contribution to the spectrum. While a more detailed elucidation of the origin of this negative contribution is beyond the scope of this study, this finding clearly demonstrates the enhanced insight into the water structures that can be gained by a direct comparison of the nonlinear vibrational responses from interfacial water at different depths.

From this qualitative analysis of the water responses from the two interfacial regions, we now turn to the quantitative analysis of the water anisotropy. Figure 5c shows the resulting reconstructed depth-dependent susceptibility $\chi_{\left(\omega_{\mathrm{IR}}\right)}^{\left(2\right)}\left(z\right)$ , which gives direct access to the evolution of the structural anisotropy of water with depth. This 3D plot reveals that the majority of the water anisotropy is clearly located in the BIL, where relatively large values for the susceptibility are obtained compared to much smaller amplitudes in the DL. The reason why the contributions from BIL and DL nevertheless yield similar depth-integrated susceptibilities (as shown in Figure 5a) originates from the very different thicknesses of water that constitute the two regions (∼1 nm for the BIL vs. 96 nm in the DL). The large discrepancy in amplitude of $\chi_{\left(\omega_{\mathrm{IR}}\right)}^{\left(2\right)}\left(z\right)$ between BIL and DL meanwhile indicates that the degree of preferential molecular orientation within these two regions must be vastly different.

More insight into the distribution of preferential water orientation with depth can be gained by analyzing the amplitude of the water signal as a function of *z*. Such a graph is depicted in Figure 5b, showing the depth-dependent peak maximum (at 3228 cm^–1^) of the water band. The graph shows how the preferential orientation slowly increases (exponentially) on approaching the interface and then undergoes a steep increase by 2 orders of magnitude at the transition from the DL to the BIL. This strong increase in preferential orientation seems, however, not sufficient to disturb the local H-bond structure (the H-bond strength and the orientational correlations of neighboring water molecules), as discussed above. This means that the extent of preferential molecular orientation even in the BIL must still be far from a highly aligned layer of dipoles because such a configuration would evidently induce significant distortion to the H-bond network. This conclusion is also clearly supported by the comparison of the amplitude of the water signal from the BIL to the signal from the C–H stretch vibrations. Despite the fact that there are roughly 10 times more water molecules in the BIL than CH_3_ groups, we observe that the signals from latter are about twice as large as the water signal. This consequently suggests that there is a significant amount of cancellation between the signals from individual water molecules, signifying that their orientational distributions must be rather broad. This means that the picture of a well-ordered water layer underneath the surface charges with a saturated alignment of molecular dipoles (as previously suggested for such systems)^28,76^ cannot be accurate for the investigated sample system. Instead, we must imagine the induced structural anisotropy as consisting of a macroscopic preference in molecular orientation where the ordering of water happens on a length-scale that is much larger than the orientational correlations in bulk water. In consequence, the deviations in thermodynamic properties of water in the interfacial region (compared to the bulk) are here clearly dominated by the lowering in entropy. Obviously, this somewhat surprising result cannot be generalized and is restricted to water in contact with the charged headgroups used in this study (phosphate) which are strongly hydrophilic. There are indications that the water structure shows more pronounced deviations in the BIL in other systems (e.g., more hydrophobic or electrode interfaces),^29,51,77–79^ however, for a comprehensive analysis further investigations are required.

Overall, this analysis demonstrates the deep insight into the anisotropic interfacial water structure that can be obtained using the SFG/DFG technique. Importantly, such analysis can be done on individual aqueous systems in one single measurement and without any assumptions on spectral line shapes or the modeling of the electric potential evolution within the BIL. Although the presented data analysis for decomposing BIL and DL spectra is based on the mathematical description of the depth-dependent response shown in eq 7, it is important to note that there are, in fact, only two assumptions that are required for an accurate decomposition, namely (i) the water anisotropy in the DL decays exponentially with the Debye length as the decay constant, and (ii) the BIL is very thin compared to the coherence length (which is ca. 47 nm) both of which are well justified. It should also be noted that even inaccuracies in the assumed thickness of the BIL would not have any impact on this decomposition and would only moderately change the scaling in the obtained susceptibilities. The main conclusions presented here would thus not be affected. This renders the method largely insusceptible to model-dependent bias, which sets it apart from other experimental approaches to address the depth-dependent nature of χ^(2)^ reported in recent publications.^22,24,26,48–50^ In this context we would like to underline the importance of an accurate and unambiguous decomposition of the raw SFG spectra into DL and BIL contributions for the analysis of interfacial water structures. Because of the depth-dependent phase shifts of interfacial SFG responses, any improper decomposition will not only intermix DL and BIL spectra but will also yield final spectra that show phase induced line-shape distortions (mixing between absorptive and dispersive line-shapes). Such phase distortions can easily be misinterpreted as being the vibrational signature of some specific structural motif that is e.g., exclusively formed in the BIL thus yielding an incorrect picture of interfacial water structures. As discussed above, our experimental approach greatly reduces the risk of such misconceptions. A more detailed discussion on the accuracy and the limitations of our approach can be found in the Supporting Information.

Therefore, the presented technique provides a very promising perspective for future studies on charged aqueous interfaces, such as the investigation of the changes in the anisotropic water structure with different charges (density or different charged headgroups) at varying salt concentrations. More generally, further possible applications of the technique include depth-resolved studies at electrochemical interfaces, the investigation of ion-specific hydration effects, and the role of interfacial water in charge transfer processes. The obtained depth-resolution of the presented technique for such studies is particularly relevant as it allows for more precise investigation of the interplay between water structure and electrochemical properties, as well as providing insights into the dynamics of hydration and ion distribution in various systems.

## Conclusion

In this work we have presented a novel spectroscopic approach for the investigation of charged aqueous interfaces and demonstrated that it provides deep insight into the depth-dependent anisotropic water structure. The measurements of water in contact with charged (phosphate headgroup) surfactants at low salinity conditions (10^–5^ M) reveal that the depth-dependent structural anisotropy of water is divided into two distinct regions; an induced preferential alignment of water dipoles in the bonded interfacial layer (BIL), followed by an exponentially decaying tail in the diffuse layer (DL). At the transition between the two regions (from DL to BIL), the molecular alignment undergoes a sudden increase by 2 orders of magnitude. Despite this large difference in structural anisotropy, the obtained vibrational spectra show that the local hydrogen-bonding network remains largely unperturbed across the entire interfacial region in the investigated system. This suggests that, even in the BIL, the induced molecular ordering is small enough that the molecular alignment can be achieved within the natural orientational degrees of freedom of the H-bond network. Furthermore, the obtained spectra do not show any indication of special structural motifs that are specifically formed in the BIL. These findings draw a picture of water in the BIL that is in its properties much more similar to bulk water than often described with the main difference being the macroscopic anisotropy in its orientational distribution. It would be very interesting to compare these results with molecular dynamics simulations of these systems to gain more detailed molecular-scale insight into the structures and dynamics of water in the BIL. Such investigation is planned in future work.

Overall, these findings establish a new paradigm for probing interfacial water, demonstrating that orientational ordering can coexist with intact hydrogen-bonding networks even under electrostatic influence. Our presented spectroscopic approach provides unprecedented depth sensitivity, paving the way for deeper insights into interfacial phenomena relevant to electrochemistry, biology, and environmental science.

## Supplementary Material



## Data Availability

Raw data will be made available upon reasonable request by contacting the corresponding author.

## Notes

A discussion of different experimental approaches for SFG/DFG measurements, additional technical details of the SFG/DFG spectrometer used in this work, the mathematical framework behind the time-domain acquisition of nonlinear vibrational spectra, details on sample preparation, additional information on the data analysis including the separation of DL and BIL spectra, a discussion on quadrupole and magnetic dipole contributions, as well as a discussion of accuracy and limitations of the presented approach, along with additional references (PDF)
