# Supplementary material for: Anisotropic Water Structure at Charged Interfaces Studied by Depth-Resolved Vibrational SFG/DFG Spectroscopy
Source: J Am Chem Soc. 2026 Apr 16;148(16):16679–89. doi: 10.1021/jacs.5c19517 (PMC13133791; doi:10.1021/jacs.5c19517)
Supplement: Supplementary file 1 [file ja5c19517_si_001.pdf]

Anisotropic Water Structure at Charged Interfaces Studied by Depth-Resolved Vibrational SFG/DFG Spectroscopy

Supporting Information

Álvaro Díaz Duque[1], Vasileios Balos[2], Martin Wolf[1], Alexander P. Fellows[1], and Martin Thämer[1*].

[1] Fritz-Haber-Institut der Max-Planck-Gesellschaft, Faradayweg 4-6, 14195, Berlin, Germany

[2] Instituto Madrileño de Estudios Avanzados en Nanociencia (IMDEA Nanociencia), 28049, Madrid, Spain

* Corresponding author

thaemer@fhi-berlin.mpg.de

(tel.): +49 (0)30 8413 5220

This supporting information provides a detailed account of the experimental and analytical procedures employed to obtain the depth-resolved vibrational spectra discussed in the main text. First, a comparison of the different experimental approaches for phase-resolved SFG spectroscopy is shown and their suitability for depth-resolved studies is discussed. This is followed by additional details on the specific implementation of the time-domain interferometric setup used in this study. This includes additional information on the optical setup, the generation of local oscillators (LOs), and the strategies used to suppress parasitic background contributions, which are essential for accurate measurements at liquid interfaces. In a subsequent section, the mathematical framework underlying the time-domain reconstruction of vibrational SFG and DFG spectra is outlined. This includes the derivation of the delay-dependent signal and its transformation into spectrally resolved, phase-sensitive responses along the infrared axis. This is followed by information on the preparation of the charged surfactant monolayers at the air-electrolyte interface, including the control of surface coverage and the monitoring of monolayer formation. In the last part, a discussion on possible contributions from electric quadrupolar and magnetic dipole sources is presented along with additional information on the data analysis procedure. Together, these sections provide the theoretical and technical basis for the depth-resolved spectroscopic analysis of interfacial water presented in the main manuscript.

A) Experimental

A1) Comparison of different SFG approaches for SFG/DFG measurements

Established SFG spectrometers typically belong to one of the three following types: i) fully narrowband frequency-domain approaches,[1,2] here a narrowband IR beam is sequentially scanned over the frequency range of interest, ii) broadband IR frequency-domain approaches, where a broadband IR is mixed with a narrowband upconversion beam,[3,4] and iii) the recently introduced fully broadband time-domain method.[5,6] These experimental approaches to measure SFG spectra are relatively established from regular SFG measurements, however, they are not

equally suitable for high precision phase-resolved SFG/DFG experiments on aqueous interfaces. Here we briefly discuss the theoretical and experimental aspects of these approaches in conjunction with such measurements.

In the frequency scanning approach, a narrowband IR beam is combined with an upconversion pulse to yield an SFG signal. This process is represented in the figure S-1a. The SFG signal has a certain spectral bandwidth that depends on the bandwidth of the visible (assuming that the IR frequency is a delta function) and an amplitude that scales with the nonlinear susceptibility at the given IR frequency. Obviously, in such a setting the spectrum of the generated nonlinear signal does not show any spectral features that relate to the vibrational resonances, instead this information is entirely encoded in its amplitude. By measuring the spectrally integrated SFG response on a single channel detector (SCD), while stepping through the infrared spectrum with the IR laser, the desired spectrum along the vibrational axis of the second-order susceptibility can be traced out (see figure S-1a). The frequency resolution obtained in the vibrational response here only depends on bandwidth of the IR beam (and obviously the step sizes), while the properties of the visible pulses are conceptually irrelevant. To obtain phase-resolved spectra with this approach, the interference of the nonlinear signal with a LO is analyzed. However, this process typically requires multiple measurements at different LO phases for each IR frequency.[7]

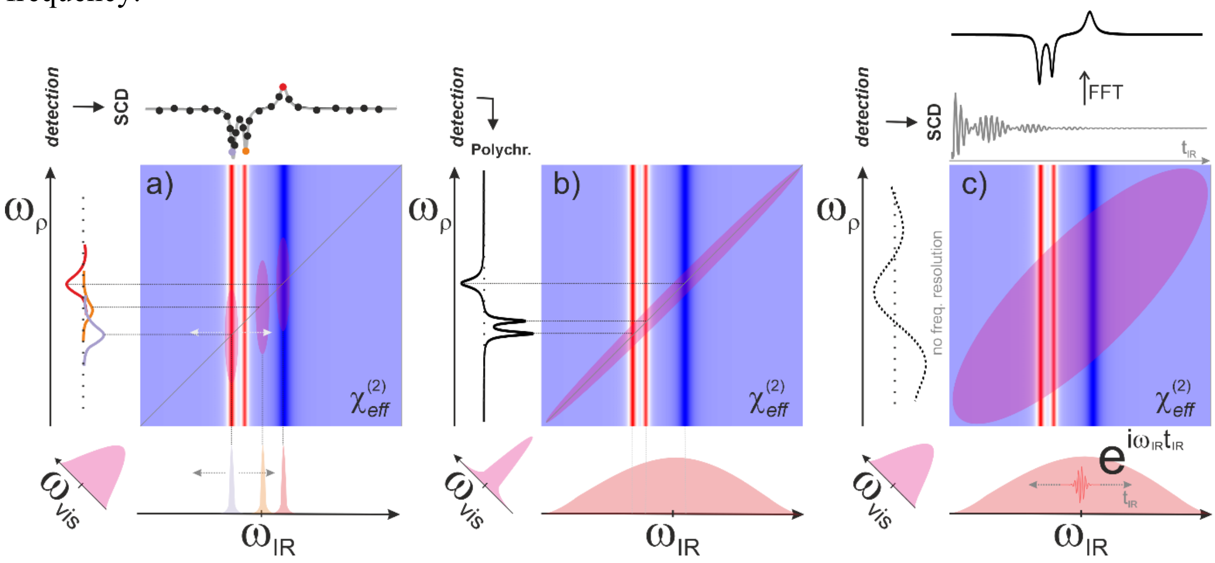

Figure S-1. Graphical representation of the three common approaches for performing frequency-resolved SFG measurements: a) frequency scanning approach, b) broadband IR frequency-domain approach, c) broadband time-domain method. For better clarity, only the imaginary parts of the susceptibility and the electromagnetic fields are shown. The vertical blue and red lines in the 2D susceptibility represent vibrational resonances while the light blue background represents the value zero.

A widely used alternative approach uses broadband IR pulses in combination with narrowband visible pulses.[3,4] The interaction of the two beams with the sample is represented in figure S-1b. It can be seen that the spectrum of the generated SFG signal now directly contains the desired vibrational information of the sample. This is exploited in these methods by spectrally resolving the generated SFG light using a polychromator. The spectrum along the vibrational axis of $\chi^{(2)}$ can then be derived by downshifting the obtained frequency axis by the center frequency of the upconversion pulse. In contrast to the first SFG approach, the frequency resolution of the vibrational response is here limited by the bandwidth of the upconversion pulse. An ideal broadband frequency domain spectrometer therefore combines a maximum

bandwidth IR pulse with a minimum bandwidth visible pulse. A significant advantage of this approach is the fact that phase-sensitive spectra can be obtained in a single measurement (except for need of a reference measurement for normalization). The LO is therefore delayed with respect to the SFG signal, giving rise to spectral interference fringes, which allow a precise determination of phase and amplitude of the resulting SFG spectrum.[3,4]

The third method is an interferometric time-domain approach employing broadband IR and broadband visible pulses (see figure S-1c), in combination with a LO.[5,8] The spectrum of the nonlinear signal does contain here the nonlinear vibrational information (all vibrational modes are excited by the broadband IR pulses), however, in contrast to the broadband frequency-domain approach, the spectral features are largely washed out by the bandwidth of the upconversion pulse. The desired frequency resolved spectrum along the vibrational axis of the two-dimensional susceptibility is here obtained using the concept of Fourier spectroscopy. By delaying the IR pulse with respect to the visible and LO pulses while detecting the intensity of the heterodyned nonlinear signal with a single channel detector, one obtains interference modulations between the generated SFG pulse and the LO. Fourier transformation of the resulting interferogram then yields the desired spectrum. The corresponding mathematical description of this time domain approach is shown in section A5.

Each method presented above can in principle be used for depth-resolved SFG/DFG measurements. However, the determination of the depth-dependent information requires enormous accuracy in phase, amplitude and frequency for both the SFG and the DFG responses, which cannot equally be achieved by the different approaches. As any deviation in one or more of these measured quantities from the correct sample responses will lead to large inaccuracies in the extracted depth profiles, it is essential that the experimental setup is adopted to these specific requirements.

The most challenging task among the requirements mentioned above is obtaining accurate and precise phase information for both the SFG and the DFG responses from a liquid sample that does not possess an atomically flat surface. The unavoidable presence of capillary waves[9] leads to constant fluctuations of the position of the phase boundary, which is also continuously influenced by the evaporation of the liquid. These spatial changes to the surface position introduce differences in the optical pathway, resulting in large phase variations of the generated nonlinear signals. An efficient way to avoid these phase fluctuations is to perform the experiment in a collinear geometry and with a LO that is generated before the sample.[6] That way the LO is linearly reflected by the same moving surface as the nonlinear signal, which leads to equal pathways for both and guarantees relative phase stability. Collinearity has the additional clear benefit that SFG and DFG signals are emitted in the same direction, which highly simplifies the interferometric measurement.

On the other hand, collinear geometries in nonlinear experiments have the clear drawback of the common appearance of parasitic signals. As all beams overlap on all optics inside the spectrometer, SFG and DFG signals can in principle be generated at all their surfaces. The use of spectral filters to remove these contributions is challenging because they would also remove the required LO. Traditionally this is overcome by avoiding spatial overlap of the beams on these optics, but this is obviously incompatible with collinearity. Another possibility is to avoid temporal pulse overlap inside the spectrometer, except at the sample surface. This can conveniently be done by introduction of dispersive material inside the beams just before the sample, which alters the delays between the infrared and upconversion pulses.[10] The

additionally resulting temporal mismatch between the generated nonlinear signals and their respective LOs can subsequently be corrected after the sample using a birefringent crystal (see A3 for details). However, for this method to work in practice, visible and IR pulses must be short in time, which is evidently incompatible with narrowband pulses, as in the first two approaches. Only the interferometric time-domain uses exclusively short pulses and is therefore best suited for the implementation of a collinear beam geometry in combination with an efficient suppression of parasitic signals.

A2) Generation of broadband infrared and vis pulses

The initial output from an amplified Ti:Sapphire laser (Astrella, Coherent), delivering (7mJ) pulses of 35fs duration at 800nm at a repetition rate of 1KHz, is split into two portions by a beam-splitter, before getting individually compressed in an internal and an external compressor. The two outputs pump two Optical Parametric Amplifiers (OPA; TOPAS Prime, Light Conversion), generating signal and idler pulses. For producing the visible output, the signal from the first OPA is isolated, and frequency doubled in a BBO crystal, yielding 690 nm light (visible). After removal of the fundamental using a short-pass filter (SPF), and subsequent power and polarization control using waveplates and polarizers, the beam (0-6μJ) is guided to the main interferometer. The mid-infrared (IR) pulses are generated by difference-frequency mixing between signal and idler outputs of the second OPA and sent to the interferometer. The resulting infrared pulse energies depend on frequency and range from 5-20μJ. For adjustment of the time-overlap of the two pulses (IR and visible), the visible beam passes an automated delay line. Once time-overlap is found, the position of this delay-stage remains fixed during the whole experiment.

A3) Generation of SFG and DFG local oscillators

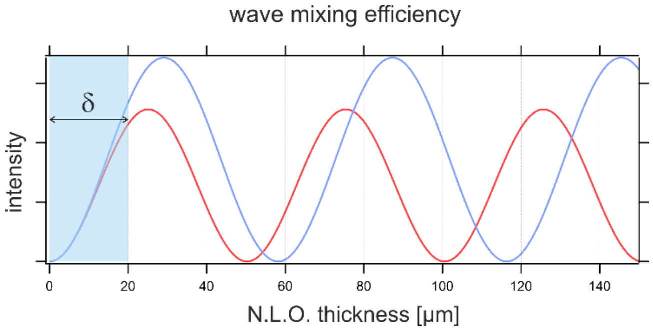

Figure S-2. Simulated SFG (red trace) and DFG (blue trace) local oscillator intensities as a function of z-cut quartz crystal thickness. The wavelengths used to calculate the curves are 690 nm for the visible beam and 3700 nm for the infrared beam.

The generation of the local oscillators (LO) for SFG and DFG is a cricial step for obtaining accurate phase resolved data with high signal-to-noise ratio. The intensity of the LO needs to generally match a relatively narrow intensity range for optimal performance of the balanced detection, which imposes important restrictions to the nonlinear material used for the LO generation. Choosing a z-cut alpha-quartz crystal has several advantages. Firstly, because of its

crystal symmetry, the polarization of the LOs can precisely be tuned by simple azimuthal rotation around its z-axis (parallel to the beam direction), as long as the IR and visible pump beams are parallelly or perpendicularly polarized. Furthermore, the response of quartz is fully nonresonant and spectroscopically flat in the desired infrared region (between 1500 and 4000 cm^{-1}).[11] However, generating both LOs in transmition in quartz comes with restrictions on the crystal thickness (δ). Due to the absence of phase-matching, the generated nonlinear signals are subject to interference effects, which highly modulate the resulting intensities as function of crystal thickness, see equation S-2

Eq. S-1

$$I(\delta) = \delta^2 \mathrm{sinc}^2\left(\frac{\Delta k_z \delta}{2}\right)$$

where Δk_z is the wavevector mismatch. This effect leads to oscillations of the generated LO intensity with crystal thickness (see figure S-2) with the oscillation period depending on the exact value for Δk_z. In principle it would be possible to maximize the LO intensity by choosing a crystal thickness at any maximum in this oscillating function, however, because of the dependency on Δk_z the positions of the intensity maxima change as function of wavelength and, more importantly, bewteen SFG and DFG. This effect can create large intensity mismatches between the two LOs with highly negative impact on the signal-to-noise ratio. The only crystal thicknesses where the SFG and DFG LO intensities can be simultaneously maximized for all desired infrared frequencies (from 1500 and 4000 cm^{-1}) is in the range between approximately 15 to 25 μm. For this reason, the z-cut quartz crystal used in our spectrometer has a thickness of 20 μm.

A4) Elimination of parasitic signals

An experimental drawback of our collinear geometry is that parasitic signals can be generated at several optics. Well-known sources of such parasitic signals are, for instance, the incoupling optics, as well as the focusing parabolic mirrors. Even though beams are only focused to generate the LO and the signal at the sample, weak parasitic signals can considerably affect the spectra from liquid interfaces. The reason for this is that aqueous interfaces normally generate extremely weak signals, and therefore the presence of parasitic signals can cause significant phase errors.

Parasitic contributions from the optics inside the interferometer generate interferograms just as does the sample response. However, these nonlinear responses from the optics are typically vibrationally nonresonant, making the resulting interferograms short (on the order of the IR autocorrelation). In contrast, the interferogram originating from the sample response can extend much further depending on the resonant spectrum, but only towards positive time delays. Therefore, the parasitic contributions can be effectively supressed by shifting the short parasitic interferograms to negative delay times, i.e. into a region outside the measured range. This can be achieved by, transmitting the beams just before the sample through a material that has a smaller group velocity at IR frequencies than at visible frequencies, such as LiF. The thickness

of the LiF window can thereby be chosen to yield sufficient time delay for a specific infrared frequency. A schematic of this precedure is shown in figure S-3.

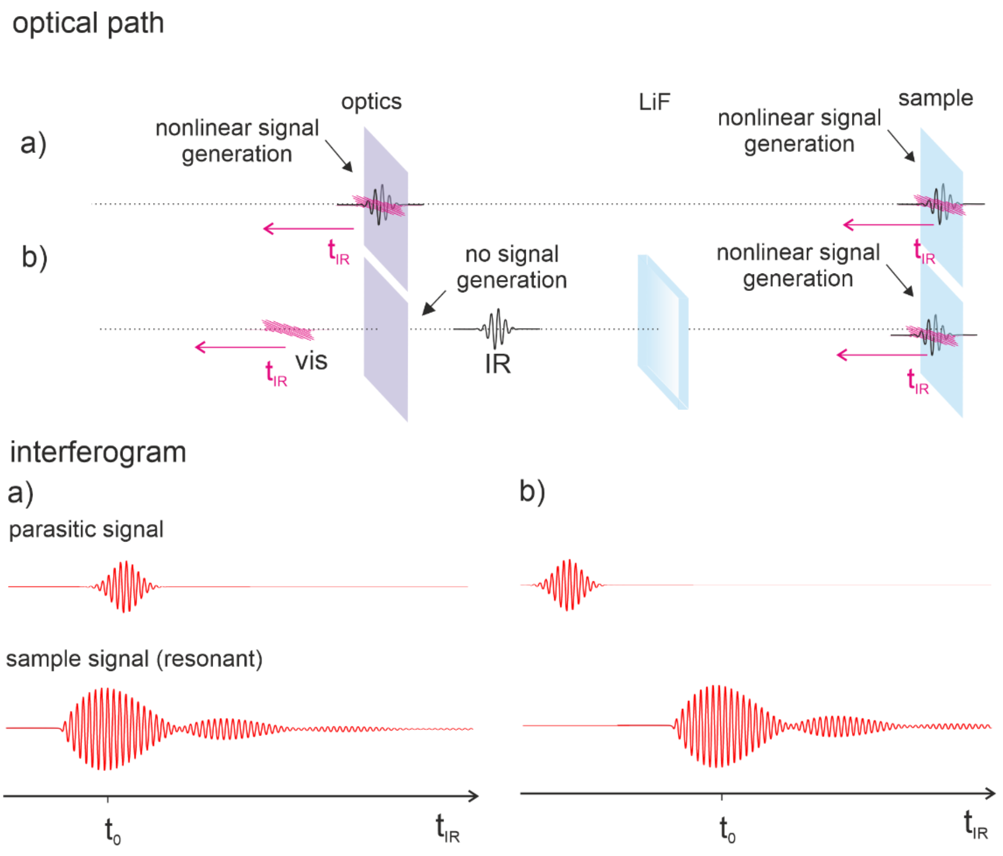

Figure S-3 Schematic representation of the optical paths (top) and resulting interferograms (bottom) a) without and b) with the introduction of a LiF window. In the latter case the interferogram arising from parasitic signal contributions generated by the optics along the beam path is shifted to negative delays by introducing a dispersive LiF window before the sample, exploiting its lower IR group velocity. This temporal displacement allows removing the parasitic signals from the measurement window.

The temporal displacement of the different pulses inside LiF, however, also affects the relative timings of the two LOs with respect to the visible pulse. Due to the dispersion curve of LiF, the SFG LO now lags behind the visible pulse whereas the DFG LOs arrives earlier. This timing mismatch between the pulses highly reduces their interference efficiency between the LOs and the nonlinear signals generated at the sample interface which significantly reduces the amplitude of the interferogram. As shown in figure S-4 this challenge is overcome by the introduction of two calcite crystals in the detection path that exploit the orthogonal polarizations of the LOs with respect to the corresponding nonlinear signals to temporally overlap them.[10]

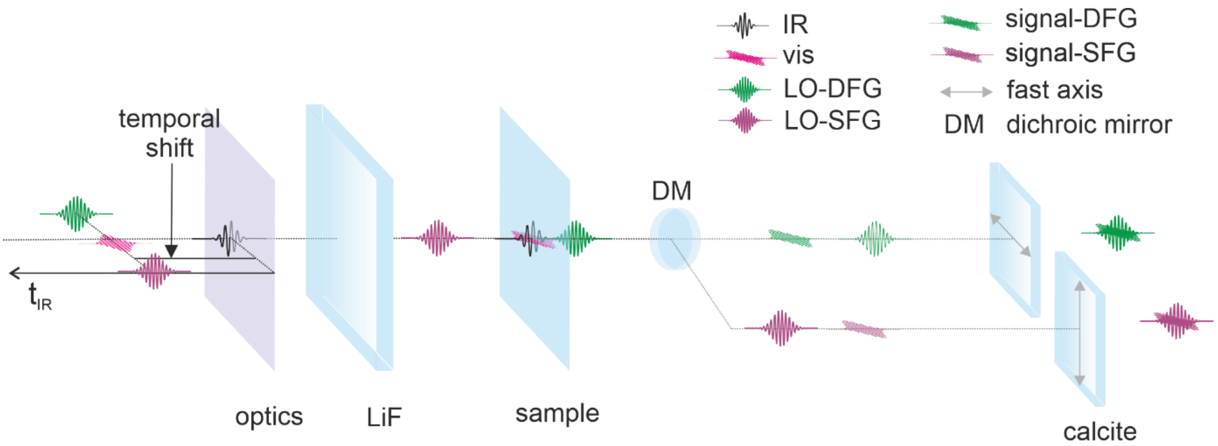

Figure S-4. Schematic showing the changes in relative timing between the different pulses while passing through the different optical ements intside the beam path. The dispersion in LiF introduces a mismatch between the visible pulse and the SFG/DFG local oscillators (LOs), reducing their interference efficiency between the LOs and the generated nonlinear signals. This mismatch is compensated downstream by a pair of birefringent calcite crystals that exploit the orthogonal polarizations of the LOs with respect to the nonlinear signals to temporally overlap them.

A5) Time-domain acquisition of nonlinear vibrational spectra

As discussed in the main text, the nonlinear vibrational response of a non-centrosymmetric medium interacting with infrared (IR) and visible laser fields, denoted as $\widetilde{E}_{(\omega_{IR})}$ and $\widetilde{E}_{(\omega_{vis})}$, respectively, can be described by

Eq. S-2

$$\widetilde{E}_{(\omega_\rho)} \propto \int_{-\infty}^{\infty} d\omega_{IR} \int_{-\infty}^{\infty} d\omega_{vis} \widetilde{E}_{(\omega_{IR})} \widetilde{E}_{(\omega_{vis})} \chi^{(2)}_{(\omega_\rho=\omega_{IR}+\omega_{vis})} \cdot \delta_{(\omega_\rho-\omega_{IR}-\omega_{vis})}$$

where $\chi^{(2)}_{(\omega_\rho=\omega_{IR}+\omega_{vis})}$ is the effective second-order susceptibility that includes both the resonant molecular response and macroscopic propagation effects as well as nonlinear Fresnel factors.

In our time-domain approach, a controlled delay t_{IR} is introduced between IR and visible pulses. This delay modulates the interaction of the visible beam with the vibrational free induction decay (FID) initiated by the IR excitation. The delayed interaction modifies the temporal evolution of the nonlinear polarization and the resulting expression for the radiated signal field becomes

Eq. S-3

$$\widetilde{E}_{(\omega_\rho,t_{IR})} \propto \int_{-\infty}^{\infty} d\omega_{IR} \int_{-\infty}^{\infty} d\omega_{vis} \widetilde{E}_{(\omega_{IR})} e^{i\omega_{IR}t_{IR}} \widetilde{E}_{(\omega_{vis})} \widetilde{E}_{(\omega_{vis})} \chi^{(2)}_{(\omega_\rho=\omega_{IR}+\omega_{vis})} \cdot \delta_{(\omega_\rho-\omega_{IR}-\omega_{vis})}$$

To access the full phase information of the nonlinear response, the signal is heterodyned with a local oscillator (LO) that is generated prior to the sample and co-propagates collinearly with the visible pulse. As a result, any temporal delay introduced between the IR and both visible pulses translates directly into a modulation of the phases between the nonlinear signal and the LO while their temporal envelopes are not affected. The resulting interference, recorded as a function of this delay, yields a time-domain interferogram that encodes both the amplitude and the phase of the effective susceptibility. This interference pattern can be expressed as

Eq. S-4

$$I_{(t_{IR})} = \int_{-\infty}^{\infty} dt\left(\mathbf{E}_{(t,t_{IR})} \cdot \mathbf{E}_{(t)}^{LO}\right) = \int_{-\infty}^{\infty} dt \int_{-\infty}^{\infty} d\omega_\rho \int_{-\infty}^{\infty} d\omega_{LO}\, \tilde{E}_{(\omega_\rho,t_{IR})} \tilde{E}_{(\omega_{LO})}^{LO} e^{i(\omega_\rho+\omega_{LO})t}$$

where $\mathbf{E}_{(t,t_{IR})}$ and $\mathbf{E}_{(t)}^{LO}$ are the electric fields of the signal and LO in the time domain. The time integration in equation S-4 leads to a vanishing contribution unless the integrand contains matching frequency components. Consequently, only terms where $\omega_\rho = -\omega_{LO}$ contribute, simplifying the expression to

Eq. S-5

$$I_{(t_{IR})} = \int_{-\infty}^{\infty} d\omega_\rho\, \tilde{E}_{(\omega_\rho,t_{IR})} \tilde{E}_{(\omega_\rho)}^{*LO}$$

Substituting equation S-3 into equation S-5 yields

Eq. S-6

$$\mathbf{I}_{(t_{IR})} = \int_{-\infty}^{\infty} d\omega_\rho \int_{-\infty}^{\infty} d\omega_{IR} \int_{-\infty}^{\infty} d\omega_{vis} \tilde{E}_{(\omega_\rho)}^{LO*} \tilde{E}_{(\omega_{IR})} e^{i\omega_{IR}t_{IR}} \tilde{E}_{(\omega_{vis})} \chi_{(\omega_\rho=\omega_{IR}+\omega_{vis})}^{(2)} \cdot \delta_{(\omega_\rho-\omega_{IR}-\omega_{vis})}$$

Fourier transformation of the interferogram then yields following expression:

Eq. S-7

$$S_{(\omega_S)} = \int_{-\infty}^{\infty} dt_{IR} \int_{-\infty}^{\infty} d\omega_\rho \int_{-\infty}^{\infty} d\omega_{IR} \int_{-\infty}^{\infty} d\omega_{vis} \tilde{E}_{(\omega_\rho)}^{LO*} \tilde{E}_{(\omega_{IR})} e^{i(\omega_{IR}-\omega_S)t_{IR}} \tilde{E}_{(\omega_{vis})} \chi_{(\omega_\rho=\omega_{IR}+\omega_{vis})}^{(2)} \cdot \delta_{(\omega_\rho-\omega_{IR}-\omega_{vis})}$$

which can be written in a more compact form as:

Eq. S-8

$$S_{(\omega_{IR})} = \int_{-\infty}^{\infty} d\omega_\rho \int_{-\infty}^{\infty} d\omega_{vis} \tilde{E}_{(\omega_\rho)}^{LO*} \tilde{E}_{(\omega_{vis})} \chi_{(\omega_\rho=\omega_{IR}+\omega_{vis})}^{(2)} \cdot \delta_{(\omega_\rho-\omega_{IR}-\omega_{vis})}$$

Here, $S_{(\omega_S)}$ was replaced by $S_{(\omega_{IR})}$ following the condition of a non-vanishing integral over ω_{IR} in equation S-7 imposing that $\omega_S = \omega_{IR}$. This step shows that the resulting spectrum is indeed a function of the vibrational frequency ω_{IR}. Overall, the expression in equation S-8 is nothing else but the projection of the shaded area onto the vibrational frequency axis in figure 1 in the main text.

For samples, where the visible and emitted fields are non-resonant, their influence on the spectral shape of the second-order response is typically negligible.[12] As a result, the susceptibility can be approximated as a function that depends only on the IR frequency, scaled by a constant pre-factor. This simplification allows the susceptibility to be factored out of the frequency integrals and expressed as $\chi_{(\omega_{IR})}^{(2)}$. Under these conditions, equation S-8 becomes

Eq. S-9

$$S_{(\omega_{IR})} \propto \chi_{(\omega_{IR})}^{(2)} \tilde{E}_{(\omega_{IR})} \int_{-\infty}^{\infty} d\omega_\rho \int_{-\infty}^{\infty} d\omega_{vis} \tilde{E}_{(\omega_\rho)}^{LO*} \tilde{E}_{(\omega_{vis})} \cdot \delta_{(\omega_\rho-\omega_{IR}-\omega_{vis})}$$

This final expression corresponds to the desired vibrational part of the susceptibility multiplied by an integral term that only depends on the complex spectra of visible and LO pulses. As the integral term is independent of $\chi^{(2)}_{(\omega_{IR})}$ it is eliminated by normalizing the sample response to a reference measurement and consequently does not affect the obtained spectra. The presence of this term, however, reveals another important aspect of the presented spectroscopic method. An alternative notation of equation S-9 is:

Eq. S-10

$$S_{(\omega_{IR})} \propto \chi^{(2)}_{(\omega_{IR})} \tilde{E}_{(\omega_{IR})} \int_{-\infty}^{\infty} dt\, \tilde{E}^{LO}_{(t)} \cdot \tilde{E}^{vis}_{(t)} \cdot e^{i\omega_{IR}t}$$

which shows that the integral term is nothing else but the Fourier transform of the product of the visible and LO fields in the time domain evaluated at the frequency ω_{IR}. This makes the amplitude of the obtained spectrum $S_{(\omega_{IR})}$ dependent on the temporal overlap between the visible and LO pulses. This dependency can be rationalized by the fact that the generated SFG pulse temporally coincides with the visible pulse which means that the above condition originates from the fact that SFG and LO pulses must temporally overlap. For best performance of the spectroscopic method in terms of signal-to-noise one should obviously maximize the amplitude of the interferogram which is why a precise matching of the arrival times of SFG and LO pulses is required. Within the presented experimental approach this is achieved by tuning the calcite crystals in the detection path presented in figure S-4.

Overall, these derivations provide the mathematical framework underlying the presented spectroscopic method. It shows how the time-domain approach, based on heterodyne detection and controlled delay between IR and visible pulses, yields phase-resolved spectra along the vibrational axis. This access to the complex spectral response forms the basis for retrieving depth-dependent structural information, as discussed in the main text.

B) Sample preparation

The sample preparation details have been described elsewhere.[13,14] Briefly, the studied samples consist of insoluble surfactant monolayers deposited at the air–electrolyte interface. Electrolyte solutions were prepared by dissolving sodium chloride (NaCl, >99% purity, Sigma-Aldrich) in deionized water (Milli-Q) to yield concentrations of 10^{-1} and 10^{-5} M.

To form charged interfaces, insoluble lipids were employed. For positively charged interfaces, we used dihexadecyldimethylammonium bromide (DHAB, >97%, Sigma-Aldrich), while for negatively charged interfaces, dihexadecyl phosphate (DHP, Sigma-Aldrich) was used. Both lipids were dissolved in chloroform to prepare spreading solutions with a concentration of 1 mg/mL. The solutions were stored at −20 °C until use.

Monolayers were prepared by dropwise deposition of the chloroform solutions onto the aqueous subphase using a micropipette, applying 2 μL per step until saturation was reached. For the presented studies it is important to work with a fully covered water surface since convection currents that are induced by heating with the infrared radiation (Marangoni convection) would otherwise drag the surfactants outside the illumination spot that is probed by our spectroscopy.[15] Saturation was identified by two simultaneous indicators. First, our fast-scan detection scheme (described in detail in the Experimental section) enables acquisition of SFG and DFG spectra

within approximately 5 seconds. This allowed for real-time tracking of the appearance of the terminal methyl (CH_3) stretching vibrations around 2800 cm^{-1}. Second, at full surface coverage, an abrupt change in surface height occurs due to the lowering in surface tension. This change in apparent surface height was monitored using a height correction system based on a position-sensitive photodiode. This setup detects small deviations in beam alignment due to vertical movement of the liquid surface. The simultaneous appearance of CH_3 vibrational signatures and a discontinuity in surface height marks the completion of monolayer formation.

C) Data treatment result section

C1) Derivation of the mathematical expression for $\chi^{(2)}_{\mathrm{eff}}$

Starting from equation 6 in the main text

Eq. S-11

$$\chi^{(2)}_{eff,(\omega_\rho=\omega_{IR}+\omega_{vis})} = \int_0^\infty \mathrm{dz}\, \mathrm{L}_{(\omega_\mathrm{p})}\mathrm{L}_{(\omega_\mathrm{vis})}\mathrm{L}_{(\omega_\mathrm{IR})}\, \chi^{(2)}(\mathrm{z})\, \mathrm{e}^{\mathrm{i}\Delta \mathrm{k_z z}}$$

we assume that the nonlinear Fresnel factors $\mathrm{L}_{(\omega)}$ are independent of z which allows for placing the factors outside the integral. We then apply the non-resonant condition for the interactions at vis, SFG and DFG frequencies ($\chi^{(2)}(\mathrm{z}) \rightarrow \chi^{(2)}_{(\omega_\mathrm{IR})}(\mathrm{z})$) and obtain

Eq. S-12

$$\chi^{(2)}_{eff,(\omega_\rho=\omega_{IR}+\omega_{vis})} = \mathrm{L}_{(\omega_\mathrm{p})}\mathrm{L}_{(\omega_\mathrm{vis})}\mathrm{L}_{(\omega_\mathrm{IR})} \int_0^\infty \mathrm{dz}\ \chi^{(2)}_{(\omega_\mathrm{IR})}(\mathrm{z})\, \mathrm{e}^{\mathrm{i}\Delta \mathrm{k_z z}}$$

In our experiments we correct all the raw data for the nonlinear Fresnel factors using the two-layer model.[16] The effective susceptibilities $\chi^{(2)}_{\mathrm{eff}}$ presented in the result section therefore correspond to

Eq. S-13

$$\chi^{(2)}_{\mathrm{eff}} = \frac{\chi^{(2)}_{eff,(\omega_\rho=\omega_{IR}+\omega_{vis})}}{\mathrm{L}_{(\omega_\mathrm{p})}\mathrm{L}_{(\omega_\mathrm{vis})}\mathrm{L}_{(\omega_\mathrm{IR})}} = \int_0^\infty \mathrm{dz}\ \chi^{(2)}_{(\omega_\mathrm{IR})}(\mathrm{z})\, \mathrm{e}^{\mathrm{i}\Delta \mathrm{k_z z}}$$

To mathematically describe the evolution of the anisotropic response of the sample with depth we assume a 2-state model adopted from the GCS theory. This assumes the presence of two distinct structural regions. Firstly, a bonded interfacial layer BIL that includes the responses of the surfactants along with the first few water layers. The thickness of the water layer within this BIL is assumed to be approximately 1nm. As the functional form of the z-dependent response within this small thickness is unknown it is described by an effective average response $\chi^{(2)}_{\mathrm{BIL}}$. The second region (diffuse layer, DL) starting below this BIL is assumed to yield responses of constant spectral shape $\chi^{(2)}_{\mathrm{DL}}$ with an amplitude that decays exponentially with z. This assumption is reasonable because this region is sufficiently far from the phase boundary to exclude the possibility of i) hydrocarbons being present (surfactants are highly insoluble in

water), and ii) significant changes in the water structure from solvation effects of the surfactant head groups. Furthermore, as the electric field in this region is relatively weak it can be expected that the water anisotropy (preferential molecular orientation) linearly follows the static electric field in that region. The nonlinear vibrational spectrum in this region should therefore resemble that of bulk water (with a small orientational anisotropy) and its amplitude should decay exponentially with the electrostatic field in z. The decay constant is meanwhile given by the Debye length following the prediction of the GCS model. With the above assumptions, we obtain the following expression for the evolution of the susceptibility with depth (Equation 7 in the main text)

Eq. S-14

$$\chi^{(2)}_{(\omega_{IR})}(z) = \begin{cases} \chi^{(2)}_{BIL} & 0 < z < z_{BIL} \\ \chi^{(2)}_{DL} \cdot e^{-(z-z_{BIL})/z_{DL}} & z \geq z_{BIL} \end{cases}$$

where $\chi^{(2)}_{DL}$ corresponds to the nonlinear response of the sample at the beginning of the DL (at $z = z_{BIL}$). Inserting equation S-14 into equation S-13 yields

Eq. S-15

$$\chi^{(2)}_{eff} = \int_0^{z_{BIL}} dz\, \chi^{(2)}_{BIL}\, e^{i\Delta k_z z} + \int_{z_{BIL}}^{\infty} dz\, \chi^{(2)}_{DL} \cdot e^{-(z-z_{BIL})/z_{DL}}\, e^{i\Delta k_z z}$$

Solving the integrals yields

Eq. S-16

$$\chi^{(2)}_{eff} = \chi^{(2)}_{BIL} \frac{1}{i\Delta k_z}\left(e^{i\Delta k_z z_{BIL}} - 1\right) + \chi^{(2)}_{DL} \frac{1}{1/z_{DL} - i\Delta k_z} e^{i\Delta k_z z_{BIL}}$$

Using the relation $|\Delta k_z z_{BIL}| \ll 1$ we find that

Eq. S-17

$$e^{i\Delta k_z z_{BIL}} \approx 1 + i\Delta k_z z_{BIL}$$

which yields

Eq. S-18

$$\chi^{(2)}_{eff} = \chi^{(2)}_{BIL}\, z_{BIL} + \chi^{(2)}_{DL}\, z_{DL} \cdot \left(\frac{1}{1 - i\Delta k_z z_{DL}} + \frac{i\Delta k_z z_{BIL}}{1 - i\Delta k_z z_{DL}}\right)$$

with $\Delta k_z z_{BIL} \ll 1$ we finally obtain

Eq. S-19

$$\chi^{(2)}_{eff} = \chi^{(2)}_{BIL}\, z_{BIL} + \chi^{(2)}_{DL}\, z_{DL} \cdot \frac{1}{1 - i\Delta k_z z_{DL}}$$

which can be transformed into

Eq. S-20

$$\chi_{\mathrm{eff}}^{(2)} = \chi_{\mathrm{BIL}}^{(2)} z_{\mathrm{BIL}} + \chi_{\mathrm{DL}}^{(2)} z_{\mathrm{DL}} \cdot \frac{1}{\sqrt{1+\Delta k_z{}^2 z_{\mathrm{DL}}{}^2}} e^{i\cdot \mathrm{atan}(\Delta k_z z_{\mathrm{DL}})}$$

This final equation equals equations 8 and 9 in the main text.

C2) Quadrupolar and magnetic dipolar contributions

The mathematical description of the nonlinear responses shown in the theory section of the main text only considers dipolar responses. However, beyond the electric dipole approximation additional electric quadrupolar and magnetic dipolar responses can in principle contribute to the measured signals. While the detailed theory of such responses in second order nonlinear spectroscopy can be found elsewhere in the literature[16,17] here we discuss possible quadrupolar and magnetic dipolar contributions to the measured spectra shown in this publication.

Electric quadrupolar and magnetic dipolar responses can roughly be divided into two categories: i) responses arising from the entire bulk which are induced by the derivative of the oscillating laser fields in the material (QB), and interfacial multipole responses arising from the derivative of the varying field amplitudes across the phase boundary (IQ). The contribution of the former is modulated by Δk_z and will therefore give rise to different amplitudes in SFG and DFG responses but in contrast to electric dipolar sources from the bulk they do not induce any phase difference.[12] However, it has been shown that such bulk quadrupolar sources must vanish in SSP polarization measurements for collinear beam geometries used in this study.[16] In consequence there should be no QB contributions present in the recorded spectra.

The source of the IQ contribution is bound to the interface and therefore does not depend on Δk_z. This suggests that IQ responses may equally contribute to SFG and DFG spectra which would make them inseparable from the desired electric dipolar responses using the presented technique. However, very recently it was found that one portion of the overall IQ term, namely a magnetic dipolar contribution $\chi_{\mathrm{MD}}^{(2)}$ shows amplitude variations for SFG and DFG responses following the relations:[17]

Eq. S-21

$$\chi_{\mathrm{MD}}^{(2)\mathrm{SFG}} \sim \frac{\omega_{DFG}}{\omega_{SFG}} \; ; \; \chi_{\mathrm{MD}}^{(2)\mathrm{DFG}} \sim \frac{\omega_{SFG}}{\omega_{DFG}} \quad \rightarrow \quad \Delta\chi_{\mathrm{MD}}^{(2)} \sim \frac{\omega_{DFG}}{\omega_{SFG}} - \frac{\omega_{SFG}}{\omega_{DFG}}$$

In consequence such interfacial magnetic dipole signals contribute differently to the SFG and DFG spectra and based on these differences, $\chi_{\mathrm{MD}}^{(2)}$ can in principle be isolated and removed from the spectra. The residual IQ contributions in contrast remain experimentally inseparable.

Indications for the presence of such magnetic dipole contributions can be found in the spectra of the DHP sample at 10^{-1} M NaCl concentration shown in the main text in figure 4. The Debye length at this ion concentration is roughly 1 nm and DHP only decorates the phase boundary which should consequently lead to almost perfectly overlapping SFG and DFG spectra similar to what has been observed for the air-water interface presented in a recent publication.[12] Closer inspection of the SFG and DFG responses presented here shows that there are small but noticeable differences which strongly suggests that these differences indeed originate from the magnetic dipole contributions described above. In figure S-5 the difference spectrum for the

DHP sample at 10^{-1} M NaCl concentration (red trace) is depicted. The spectrum shows that the deviations between SFG and DFG spectra mainly concern the C-H stretching region (mainly the pronounced peak at 2950 cm^{-1}) and must therefore originate from the DHP monolayer, more specifically from the hydrocarbon chains. In consequence, the same magnetic dipole response should appear in the measurement of the DHP sample at 10^{-5} M NaCl concentration. In figure S-5 the difference spectrum between SFG and DFG responses for latter measurement is shown (green trace) which confirms that the same spectral features in the CH region are indeed present along with the broad and phase shifted water response from the DL. With this data in hand the magnetic dipole contribution to the difference spectrum can be removed by simple subtraction of the 10^{-1} M from the 10^{-5} M trace as in the figure (black trace).

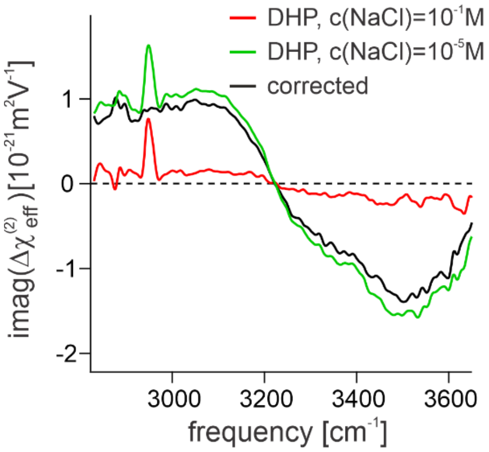

Figure S-5. SFG DFG difference spectra for DHP, c(NaCl) = 10^{-1}M (red trace) and DHP, c(NaCl) = 10^{-5}M (green trace). Black trace corresponds to the difference between the green and the red curves.

At this point it is important to note that the spectrum in figure S-5 (red trace) is the contribution of the magnetic dipole responses to the SFG/DFG difference spectra $\Delta\chi^{(2)}_{\mathrm{MD}}$ and not the contribution to the measured raw spectra ($\chi^{(2)\mathrm{SFG}}_{\mathrm{MD}}$ and $\chi^{(2)\mathrm{DFG}}_{\mathrm{MD}}$, respectively). Latter can be obtained based on equations S-21 via:

Eq. S-22

$$\chi^{(2)\mathrm{SFG}}_{\mathrm{MD}} = \frac{\Delta\chi^{(2)}_{\mathrm{MD}}}{1-\frac{\omega_{SFG}^2}{\omega_{DFG}^2}} \quad \text{and} \quad \chi^{(2)\mathrm{DFG}}_{\mathrm{MD}} = \frac{\Delta\chi^{(2)}_{\mathrm{MD}}}{\frac{\omega_{DFG}^2}{\omega_{SFG}^2}-1}$$

The resulting contributions for SFG and DFG are shown in figure 5 in the main text.

Besides the apparent magnetic dipole contribution additional signals from interfacial electric quadrupole sources can obviously contribute to the measured spectra. However, similar to the observed magnetic dipole signals they can be expected to be rather small and since they yield precisely equal SFG and DFG spectra they would exclusively contribute to the extracted BIL spectra.

C2) Determination of DL and BIL spectra

For the determination of the DL spectrum, we use the difference spectrum $\Delta\chi_{\mathrm{eff}}^{(2)}$ (difference between SFG and DFG spectra) where the magnetic dipole contribution is removed (black trace figure S-5). Using equation 10 from the main text we can obtain the DL spectrum as follows

Eq. S-23

$$\chi_{\mathrm{DL}}^{(2)} z_{\mathrm{DL}} = \frac{\Delta\chi_{\mathrm{eff}}^{(2)}}{\left(C_{\mathrm{SFG}}(z_{\mathrm{DL}}, \Delta k_z^{\mathrm{SFG}}) \cdot e^{i\cdot \mathrm{atan}(\Delta k_z^{\mathrm{SFG}} \cdot z_{\mathrm{DL}})} - C_{\mathrm{DFG}}(z_{\mathrm{DL}}, \Delta k_z^{\mathrm{DFG}}) \cdot e^{i\cdot \mathrm{atan}(\Delta k_z^{\mathrm{DFG}} \cdot z_{\mathrm{DL}})}\right)}$$

where $\chi_{\mathrm{DL}}^{(2)} z_{\mathrm{DL}} = \int_{z_{BIL}}^{\infty} dz\, \chi_{\mathrm{DL}}^{(2)}(z)$ (this spectrum is shown in figure 6a in the main text).

The effective contribution of the DL to the SFG and DFG spectra are obtained using following expression

Eq. S-24

$$\chi_{\mathrm{eff,DL}}^{(2)} = \chi_{\mathrm{DL}}^{(2)} z_{\mathrm{DL}} \cdot C_{\mathrm{SFG}}(z_{\mathrm{DL}}, \Delta k_z) \cdot e^{i\cdot \mathrm{atan}(\Delta k_z \cdot z_{\mathrm{DL}})}$$

with the respective values for Δk_z for SFG and DFG, respectively. The resulting spectra are shown in figure 5 (main text).

The BIL contribution is then obtained by subtracting the effective DL contribution from the measured raw data (either from the SFG or the DFG data). Note, in contrast to the DL spectrum the BIL spectrum now still contains the magnetic dipole contribution, $\chi_{\mathrm{MD}}^{(2)}$ which is removed by simple subtraction. The final BIL spectrum ($\chi_{\mathrm{BIL}}^{(2)} z_{BIL} = \int_0^{z_{BIL}} dz\, \chi_{\mathrm{BIL}}^{(2)}(z)$) is shown in figure 6a in the main text.

At this point, it is important to discuss a few conceptual points about the data analysis presented here that are essential for a correct interpretation of the obtained spectra. Taking the difference between SFG and DFG responses isolates the spectral contributions that originate from extended depths. This difference spectrum therefore exclusively contains information about the DL provided that the BIL is very thin compared to the coherence length (ca. 47 nm for our beam geometry). These contributions are then modeled as an exponentially decaying function in z which allows for determination of the intrinsic spectral shape and amplitude of $\chi_{\mathrm{DL}}^{(2)}$ using equation S-23. The only parameters that are needed to perform this step are the Δk_z values for SFG and DFG and the Debye length z_{DL}, all of which can be accurately calculated. Importantly, no assumptions must be made about the spectral shape or the amplitude scaling of the DL contribution as this information is contained in the difference spectrum. As shown above, the spectrum of the BIL is then obtained by subtracting the effective DL contribution ($\chi_{\mathrm{eff,DL}}^{(2)}$, equation S-24) from either the SFG or the DFG raw spectra. Within this method, the obtained spectrum, and thus the quantity $\chi_{\mathrm{BIL}}^{(2)} z_{BIL}$, then corresponds to the deviation of the acquired overall spectra from a purely exponentially decaying DL contribution. The deviation can thereby have two origins: i) deviations in the amplitude evolution with z from a mono-exponential function close to the interface and ii) spectral changes. In the data presented in the main text both effects are present. Spectral changes are observed for the CH resonances which are absent in the DL spectra while the presence of the strong water signal in the BIL spectrum

shows that the preferential molecular water alignment in the BIL highly surpasses the amount that would correspond to a mono-exponential function.

Importantly, up to this point, no assumption for the thickness of the BIL (z_{BIL}) is needed as long as it is small enough to not contribute to the SFG/DFG difference spectrum, which means that phase shifts between SFG and DFG responses originating from the light propagation within the BIL can be neglected. Consequently, we can summarize the two assumptions that are required for the entire described method to work accurately: i) the DL signal has a constant spectral shape with an amplitude that decays exponentially with depth (with the Debye length as decay constant), and ii) the BIL is thin enough that depth-related phase effects can be neglected. However, the above discussion also shows that the method works best if there is a large difference between the thickness of the BIL and the decay constant in the DL i.e. for solutions with relatively low salt concentrations. For high concentrations, the Debye length gets so short that the difference between SFG and DFG spectra nearly vanishes (see e.g. the 10^{-1} M cases in figure 3 in the main text). In consequence, the difference spectrum is then dominated by noise which reduces the accuracy of the decomposition. On the other hand, for high ionic strengths, BIL and DL merge to an increasing extent such that their separation is even conceptually little meaningful. Furthermore, it is important to point out that our method does not yield any information on the structural evolution of the water with depth inside the BIL as it only yields the effective, depth-integrated response of the BIL. In other words, while it does allow for drawing conclusions on the H-bond network within this region (a few water layers) by analyzing the obtained spectral line shape (as shown in the main text), the spectral evolution with depth on a molecular scale is inaccessible.

In the last step shown in figure 5c in the main text, the depth-dependent susceptibility is reconstructed from the BIL and DL spectra. For this step, an additional parameter is required, namely the thickness of the bonded interfacial layer z_{BIL}. Here we assume the thickness to be approximately 1 nm. The division of the BIL spectrum by this thickness then yields the average spectrum (susceptibility) inside the BIL as shown in figure 5c. This step obviously contains some ambiguity since the exact thickness of the BIL is not known (1 nm should be an accurate estimate). However, it is important to note that the choice of a value for z_{BIL} has no significant impact on the analysis presented in the main text. Since a knowledge of the BIL thickness is not required for the decomposition of the overall spectra into BIL and DL spectra (as shown above), z_{BIL} exclusively acts as a simple scaling factor for the conversion of the integrated BIL spectrum into the average susceptibility. That means that any inaccuracy in the value for z_{BIL} used in this work only translates into a corresponding inaccuracy in the amplitude scaling of the BIL susceptibility in figure 5c). As the susceptibilities in BIL and DL are found to deviate by two orders of magnitude, such relatively small potential inaccuracies are not significant for the presented comparison and do not have any impact on the conclusions drawn in this work.

References

(1) Shen, Y. R. Surfaces Probed by Nonlinear Optics. *Surf. Sci.* **1994**, *299/300*, 551–562.

(2) Ostroverkhov, V.; Waychunas, G. A.; Shen, Y. R. New Information on Water Interfacial Structure Revealed by Phase-Sensitive Surface Spectroscopy. *Phys. Rev. Lett.* **2005**, *94* (4), 2–5.

(3) Richter, L. J.; Petralli-Mallow, T. C.; Stephenson, J. C. Vibrationally Resolved Sum-Frequency

Generation with Broad-Bandwidth Infrared Pulses. *Opt. Lett.* **1998**, *23* (20), 1594–1596.

(4) Funk, S.; Bonn, M.; Denzler, D. N.; Hess, C.; Wolf, M.; Ertl, G. Desorption of CO from Ru (001) Induced by near-Infrared Femtosecond Laser Pulses. *J. Chem. Phys.* **2000**, *112* (22), 9888–9897.

(5) Laaser, J. E.; Xiong, W.; Zanni, M. T. Time-Domain SFG Spectroscopy Using Mid-IR Pulse Shaping: Practical and Intrinsic Advantages. *J. Phys. Chem. B* **2011**, *115* (11), 2536–2546.

(6) Thämer, M.; Campen, R. K.; Wolf, M. Detecting Weak Signals from Interfaces by High Accuracy Phase-Resolved SFG Spectroscopy. *Phys. Chem. Chem. Phys.* **2018**, *20* (40), 25875–25882.

(7) Xu, X.; Shen, Y. R.; Tian, C. Phase-Sensitive Sum Frequency Vibrational Spectroscopic Study of Air/Water Interfaces: H2O, D2O, and Diluted Isotopic Mixtures. *J. Chem. Phys.* **2019**, *150* (14).

(8) Balos, V.; Garling, T.; Díaz Duque, Á.; John, B.; Wolf, M.; Thämer, M. Phase-Sensitive Vibrational Sum and Difference Frequency-Generation Spectroscopy Enabling Nanometer-Depth Profiling at Interfaces. *J. Phys. Chem. C* **2022**, *126* (26), 10818–10832.

(9) Perlin, M.; Schultz, W. W. Capillary Effects on Surface Waves. *Annu. Rev. Fluid Mech.* **2000**, *32*, 241–274.

(10) Garling, T.; Campen, R. K.; Wolf, M.; Thämer, M. A General Approach to Combine the Advantages of Collinear and Noncollinear Spectrometer Designs in Phase-Resolved Second-Order Nonlinear Spectroscopy. *J. Phys. Chem. A* **2019**, *123* (51), 11022–11030.

(11) Thämer, M.; Garling, T.; Campen, R. K.; Wolf, M. Quantitative Determination of the Nonlinear Bulk and Surface Response from Alpha-Quartz Using Phase Sensitive SFG Spectroscopy. *J. Chem. Phys.* **2019**, *151*, 064707.

(12) Fellows, A. P.; Díaz Duque, Á.; Balos, V.; Lehmann, L.; Netz, R. R.; Wolf, M.; Thämer, M. How Thick Is the Air-Water Interface? - A Direct Experimental Measurement of the Decay Length of the Interfacial Structural Anisotropy. *Langmuir* **2024**, *40*, 18760–18772.

(13) Nagata, Y.; Hasegawa, T.; Backus, E. H. G.; Usui, K.; Yoshimune, S.; Ohto, T.; Bonn, M. The Surface Roughness, but Not the Water Molecular Orientation Varies with Temperature at the Water-Air Interface. *Phys. Chem. Chem. Phys.* **2015**, *17* (36), 23559–23564.

(14) Fellows, A. P.; Lehmann, L.; Díaz Duque, Á.; Wolf, M.; Netz, R. R.; Thämer, M. The Importance of Layer-Dependent Molecular Twisting for the Structural Anisotropy of Interfacial Water. *arXiv:2505.12962* **2025**, 1–26.

(15) Fellows, A. P.; Casford, M. T. L.; Davies, P. B. Investigating Bénard-Marangoni Migration at the Air-Water Interface in the Time Domain Using Sum Frequency Generation (SFG) Spectroscopy of Palmitic Acid Monolayers. *J. Chem. Phys.* **2022**, *156* (16).

(16) Morita, A. *Theory of Sum Frequency Generation Spectroscopy*; Springer Nature Singapore, 2018.

(17) Lehmann, L.; Becker, M. R.; Tepper, L.; Fellows, A. P.; Díaz Duque, Á.; Thämer, M.; Netz, R. R. Beyond the Electric Dipole Approximation: Electric and Magnetic Multipole Contributions Reveal Biaxial Water Structure from SFG Spectra at the Air-Water Interface. *arXiv:2505.19856* **2025**, 1–14.
